# A postnatal molecular switch drives activity-dependent maturation of parvalbumin interneurons

**DOI:** 10.1016/j.cell.2025.06.029

**Published:** 2025-07-15

**Authors:** Monika Moissidis, Leyla Abbasova, Martijn Selten, Rafael Alis, Clémence Bernard, Yaiza Domínguez-Canterla, Fazal Oozeer, Shenyue Qin, Audrey Kelly, Laura Mòdol, Navneet A. Vasistha, Benjamin Jones, Pawan Dhami, Konstantin Khodosevich, Fursham Hamid, Paul Lavender, Nuria Flames, Oscar Marín

**Affiliations:** 1 Centre for Developmental Neurobiology, Institute of Psychiatry, Psychology and Neuroscience, King’s College London, London SE1 1UL, UK; 2 Medical Research Council Centre for Neurodevelopmental Disorders, King’s College London, London SE1 1UL, UK; 3 Developmental Neurobiology Unit, Instituto de Biomedicina de Valencia IBV- CSIC, Valencia 46012, Spain; 4 Valencia Biomedical Research Foundation, Centro de Investigación Príncipe Felipe (CIPF), Associated Unit to the Instituto de Biomedicina de Valencia (IBV), Valencia 46012, Spain; 5 Peter Gorer Department of Immunobiology, School of Immunology and Microbial Science, King’s College London, London SE1 9RT, UK; 6 King’s Centre for Lung Health, King’s College London, London SE1 9RT, UK; 7 Biotech Research and Innovation Center (BRIC), Copenhagen Biocenter, Faculty of Health and Medical Sciences, University of Copenhagen, Copenhagen 2200, Denmark; 8 Genomics Research Platform and Single Cell Laboratory, Guy’s Hospital, London SE1 9RT, UK

## Abstract

Cortical neurons are specified during embryonic development but often acquire their mature properties at relatively late stages of postnatal development. This delay in terminal differentiation is particularly prominent for fast-spiking parvalbumin-expressing (PV^+^) interneurons, which play critical roles in regulating the function of the cerebral cortex. We found that the maturation of PV^+^ interneurons is triggered by neuronal activity and mediated by the transcriptional cofactor peroxisome proliferator-activated receptor-gamma coactivator 1-alpha (PGC-1*α*). Developmental loss of PGC-1*α* prevents PV^+^ interneurons from acquiring unique structural, electrophysiological, synaptic, and metabolic features and disrupts their diversification into distinct subtypes. PGC-1*α* functions as a master regulator of the differentiation of PV^+^ interneurons by directly controlling gene expression through a transcriptional complex that includes ERRγ and Mef2c transcription factors. Our results uncover a molecular switch that translates neural activity into a specific transcriptional program, promoting the maturation of PV^+^ interneurons at the appropriate developmental stage.

## Graphical abstract

**Graphical abstract:**
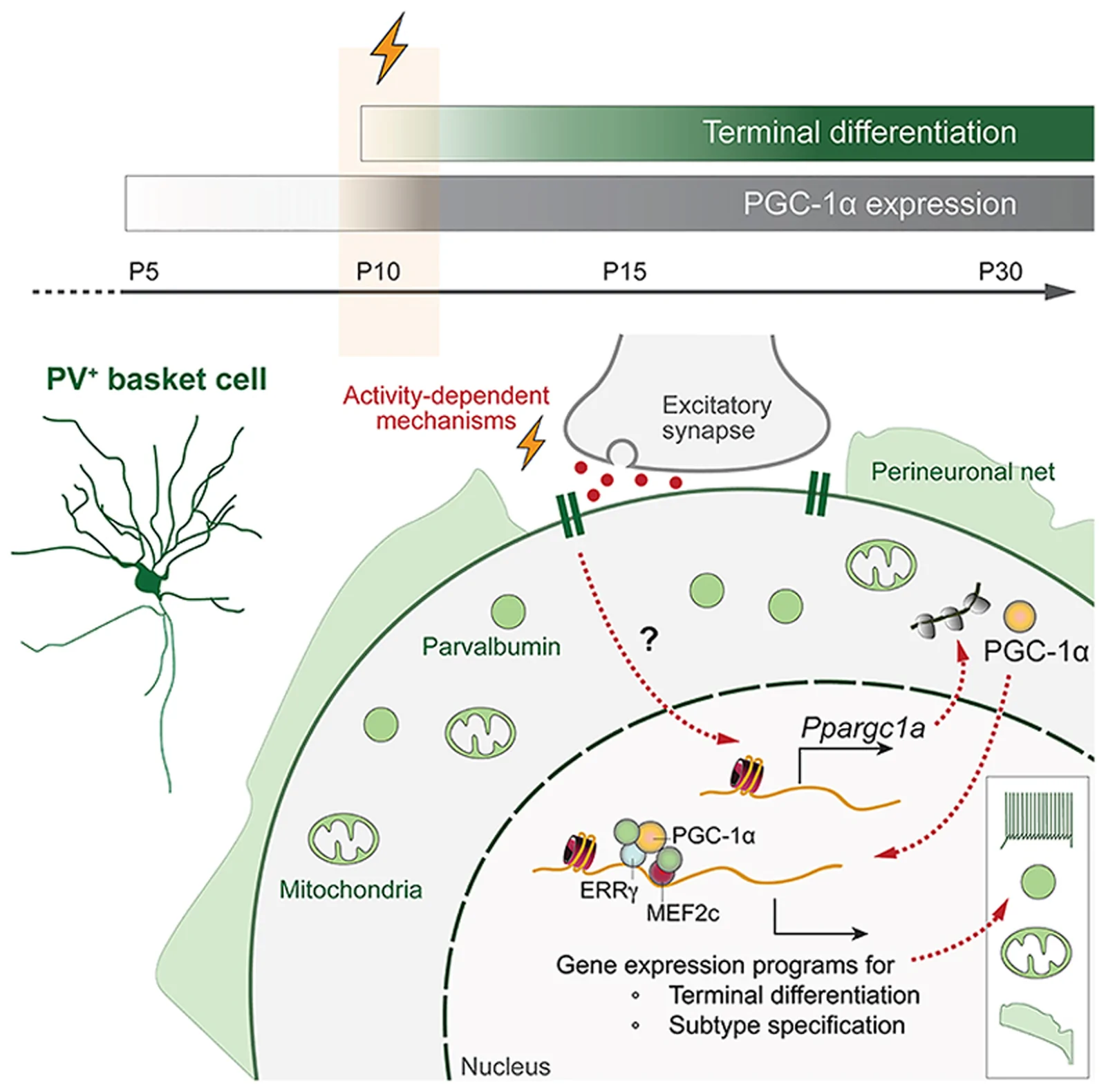

## Introduction

Understanding the molecular mechanisms controlling the diver-sification and terminal differentiation of neurons in the mammalian nervous system remains an immense challenge. This task is particularly daunting in the mammalian cerebral cortex, which contains a vast diversity of glutamatergic principal neurons and GABAergic interneurons.^1–3^ Cortical neurons are specified during embryonic development,^4–7^ but many do not express genes that regulate critical aspects of their function until relatively late stages of postnatal development. This observation suggests that the terminal differentiation of cortical neurons is linked to neuronal activity and the environmental signals animals receive during postnatal development, as recently shown for certain types of excitatory cells.^8^ However, since the maturation of cortical neurons is asynchronous, different cell types must have evolved specific molecular mechanisms for translating environmental cues into signals for terminal differentiation at the appropriate developmental stage.

Fast-spiking interneurons expressing the calcium-binding protein parvalbumin (PV^+^) are critical regulators of cortical function.^9,10^ They have unique electrophysiological characteristics associated with high metabolic demands, including fast action potential (AP) kinetics and ion conductances.^10^ Most PV^+^ basket cells are wrapped by perineuronal nets, a condensed form of the extracellular matrix that protects neurons from oxidative stress^11^ and regulates their plasticity.^12^ Critically, PV^+^ interneurons make synapses on the somatic and perisomatic compartments of pyramidal cells, exerting firm control over their firing.^10^ This is required to generate gamma oscillations,^13–15^ a high-frequency network rhythm essential for multiple cognitive operations disrupted in neuropsychiatric disorders.^16^

Like other cells, PV^+^ interneurons acquire unique morphological, neurochemical, and electrophysiological features through the progressive selection of dedicated gene expression programs during development.^4,5,17^ However, PV^+^ interneurons exhibit a particularly protracted period of maturation compared with other interneurons. In humans, for example, the expression of PV only increases dramatically after infancy, and the perineuronal nets covering these cells acquire their mature appearance at around 8 years old.^18,19^ Even in rodents, which exhibit a much more accelerated tempo of neuronal differentiation than primates,^20,21^ PV^+^ interneurons only fully acquire the properties that enable their high-frequency APs well into the third week of postnatal life.^22,23^ The maturation of PV^+^ interneurons critically regulates the transition of cortical activity from highly synchronous to sparse and decorrelated,^24^ the closure of critical periods of heightened plasticity,^25–27^ and the developmental emergence of complex cognitive abilities in mammals.

The maturation of PV^+^ interneurons occurs parallel to a rapid increase in glutamatergic synapses received by these cells,^28–30^ suggesting that neuronal activity plays a role in this process. Consistent with this hypothesis, the density of glutamatergic synapses targeting PV^+^ interneurons in the adult cortex predicts cellular PV levels in mice and humans,^31,32^ and sensory experience promotes the maturation of PV^+^ cells.^33–36^ Neuronal activity is also responsible for the secretion of brain-derived neurotrophic factor (BDNF) by excitatory neurons, which supports PV^+^ interneuron development.^37–39^ However, the precise mechanisms through which neuronal activity may trigger this critical process are unknown.

Here, we investigated the cellular and molecular mechanisms regulating the terminal differentiation of PV^+^ interneurons in the mouse cerebral cortex. We found that the maturation of PV^+^ interneurons is modulated by neuronal activity and that this process requires the function of peroxisome proliferator-activated receptor-gamma coactivator 1-alpha (PGC-1*α*), a transcriptional cofactor upregulated by prospective PV^+^ interneurons during postnatal development. In other tissues, different isoforms of PGC-1*α* are involved in adaptive responses to change, a function that is thought to be mediated by their capacity to promote mitochondrial biogenesis and energy metabolism.^40,41^ PGC-1*α* regulates gene expression in pyramidal cells and interneurons in the adult cortex,^42–44^ but its role in the protracted differentiation of PV^+^ interneurons has not been explored. Our experiments demonstrate that PGC-1*α* is an activity-dependent master regulator of the maturation of PV^+^ interneurons.

## Results

### Activity-dependent regulation of PV and WFA expression

In the primary somatosensory cortex (S1), the expression of PV and *Wisteria floribunda* Agglutinin (WFA), a marker of perineuronal nets, is first detected at P10 (Figures S1A and S1B). It increases sharply between P10 and P12 and then rises steadily until P21 (Figures S1A and S1B). By contrast, the fast-spiking properties of PV^+^ interneurons mature at a slower pace.^28,30^ For instance, while there is a prominent increase in PV and WFA expression levels between P12 and P13 (Figures S1C and S1D), no differences can be detected in the AP kinetics and maximum firing frequency of PV^+^ interneurons at these stages (Figure S1E; Table S1). The slow functional maturation of these cells reflects the protracted expression of critical proteins such as the potassium voltage-gated channels Kv1.1 and Kv3.1 (Figure S1F). Consequently, measuring PV and WFA levels is the most reliable approach to monitor the maturation of these cells during early postnatal development.

**Figure 1:**
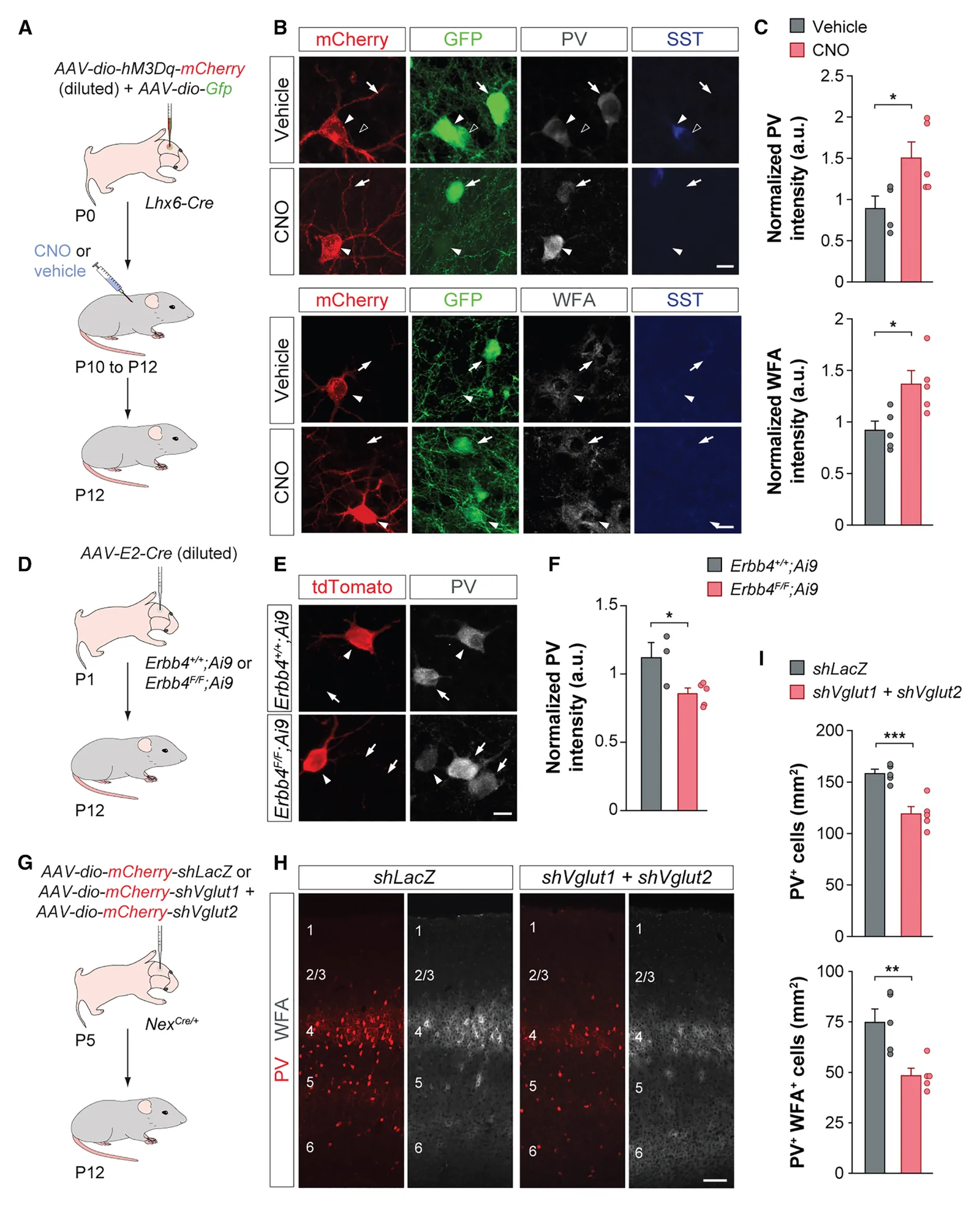
PV and WFA expression in prospective PV^+^ interneurons depends on neuronal activity during early postnatal development (A) Experimental design. (B) PV and WFA expression in S1 L2/3 prospective PV^+^ cells (recognized by the absence of SST expression) expressing mCherry or GFP in control (vehicle) and experimental (CNO) conditions at P12. Arrows point at GFP^+^ PV^+^ cells, arrowheads point at mCherry^+^ PV^+^ cells, and open arrowheads point at GFP^+^ SST^+^ cells. (C) PV and WFA levels in S1 L2/3 mCherry^+^ prospective PV^+^ cells normalized by PV and WFA levels in neighboring mCherry^-^ GFP^+^ prospective PV^+^ cells at P12. (D) Experimental design. (E) PV expression in S1 L5/6 tdTomato^+^ PV^+^ cells in *ErbB4^+/+^;Ai9* and *ErbB4^F/F^;Ai9* mice at P12. Arrows and arrowheads point to uninfected and infected cells, respectively. (F) PV levels in S1 L5/6 infected cells normalized by PV levels in neighboring uninfected cells at P12. (G) Experimental design. (H) PV and WFA expression in coronal sections through S1 from control (*shLacZ*) and experimental (*shVglut1 + shVglut2*) mice at P12. (I) Density of PV^+^ and PV^+^ WFA^+^ cells across all layers in S1 from control (*shLacZ*) and experimental (*shVglut1* + *shVglut2*) mice at P12. Data are presented as mean ± SEM. Scale bars, 10 μm (B and E) and 100 μm (H). *p < 0.05, **p < 0.01, ***p < 0.001. See Table S5 for details on statistics. Related to Figures S1 and S2.

To investigate whether the onset of PV expression and perineuronal net formation is modulated by neuronal activity, we increased the activity of prospective PV^+^ interneurons using a chemogenetic approach based on designer receptors exclusively activated by designer drugs (DREADDs).^45^ In brief, we injected S1 of postnatal day (P)0 *Lhx6-Cre* pups with a cocktail of Cre-dependent adeno-associated viruses (AAVs) expressing GFP and mutant G-protein-coupled receptors that induce neuronal activation (hM3Dq, fused to mCherry) following the administration of the pharmacologically inert molecule clozapine-*N*-oxide (CNO). We used *Lhx6-Cre* mice because cortical interneurons derived from the medial ganglionic eminence (MGE), i.e., prospective PV^+^ interneurons and somatostatin-expressing (SST^+^) interneurons, express this transcription factor as soon as they become postmitotic.^46^ Since SST is expressed by migrating SST^+^ interneurons,^47^ prospective PV^+^ interneurons can be identified as GFP^+^ (recombined) cells lacking SST expression. We titrated the hM3Dq virus to infect a small fraction of interneurons in each animal (Figure S2A), thereby minimizing network-wide effects (Figure S2B). We then injected CNO or vehicle twice daily from P10 to P12 and analyzed the impact of this manipulation at P12 (Figure 1A). We verified that CNO activates interneurons expressing hM3Dq and does not affect the intensity of PV and WFA in interneurons lacking hM3Dq expression (Figures S2C and S2D). We found that the intensity of PV and WFA is higher in prospective layer (L)2/3 PV^+^ interneurons expressing hM3Dq than in neighboring interneurons lacking hM3Dq expression (GFP^+^ mCherry^–^) (Figures 1B and 1C). We observed similar results in experiments where hM3Dq expression was excluded from SST^+^ interneurons by injecting Cre^ON^/Flp^OFF^ hM3Dq-mCherry AAVs in *Lhx6-Cre;Sst^Flp/+^* mice (Figures S2E–S2G), demonstrating that direct activation of PV^+^ interneurons promotes the expression of PV and WFA.

We wondered whether reducing the activity of PV^+^ interneurons had the opposite effect on PV expression. To test this hypothesis, we sparsely infected Lhx6^+^ interneurons by injecting S1 of P0 *Lhx6-Cre* pups with a cocktail of Cre-dependent AAVs expressing GFP and mutant G-protein-coupled receptors that reduce neuronal activation (hM4Di) following CNO administration. We then injected CNO or vehicle twice daily from P10 to P12 and analyzed the impact of this manipulation at P12 (Figure S2H). We focused on L5 because PV^+^ interneurons in this layer are at a more advanced stage of maturation than in L2/3 at this age. We found that PV intensity is decreased in prospective PV^+^ interneurons expressing hM4Di compared with neighboring GFP^+^ cells lacking hM4Di expression (Figures S2I and S2J). Together, these experiments suggested that neuronal activity is necessary and sufficient to induce PV and WFA expression in developing PV^+^ interneurons.

Since pyramidal cells are the primary source of excitatory inputs for cortical interneurons, we investigated whether the formation of glutamatergic synapses modulates the expression of PV and WFA in developing PV^+^ interneurons. To this end, we manipulated the density of glutamatergic synapses contacting prospective PV^+^ interneurons during early postnatal development. We and others have previously shown that the formation of these synapses requires the tyrosine kinase receptor ErbB4.^48–50^ To delete ErbB4 in a sparse population of prospective PV^+^ interneurons, we crossed *Erbb4^+/+^* and *Erbb4^F/F^* mice with *RCL^tdTomato/+^* (*Ai9*) reporter mice to label cells with tdTomato following Cre-mediated recombination. We then injected S1 of P1 pups with AAVs expressing Cre under the control of an enhancer that directs Cre expression to putative PV^+^ interneurons^51^ (Figure 1D). We titrated the virus to infect a small fraction of interneurons (Figure S2K) and verified that ErbB4 loss reduced the density of excitatory inputs contacting infected PV^+^ interneurons by P12 (Figure S2L). We found that tdTomato^+^ PV^+^ interneurons in *Erbb4^F/F^;Ai9* mice had lower levels of PV intensity than neighboring uninfected PV^+^ cells (Figures 1E and 1F). These experiments reinforced the notion that neuronal activity, mediated by glutamatergic synapses, modulates the onset of PV expression.

To further support this idea, we decreased cortical glutamatergic neurotransmission using conditional short-hairpin RNA (shRNA) vectors against *Slc17a7* and *Slc17a6*, which encode Vglut1 and Vglut2, respectively. We injected Cre-dependent AAVs expressing the shRNA constructs into S1 of P5 *Nex^Cre/+^* pups, in which Cre is expressed by principal cells (Figure 1G). We confirmed the ability of *shVglut1* and *shVglut2* to downregulate the expression of the corresponding target genes (Figures S2M and S2N). We timed injections to avoid the period of programmed cell death in interneurons, which is dependent on glutamatergic neurotransmission,^52–54^ and verified that Vglut1 and Vglut2 downregulation did not impact the density of Lhx6^+^ cells at P12 (Figure S2O). By contrast, the density of cells expressing detectable levels of PV and perineuronal nets is decreased in these mice compared with controls (Figures 1H, 1I, and S2P). Altogether, our experiments indicated that cortical excitatory activity is critical for the onset of PV and WFA expression in PV^+^ interneurons.

Pyramidal cells are not the only source of excitation for cortical PV^+^ interneurons. Thalamocortical projections from the ventral posteromedial nucleus of the thalamus contact L4 PV^+^ interneurons from P7 onward,^55–57^ and whisker stimulation at P12 modulates the impact of PV^+^ interneurons onto local cortical networks.^58^ To examine whether thalamocortical activity modulates the onset of PV expression in L4, we injected a cocktail of Cre-dependent AAVs expressing *mCherry-shVglut1* and *mCherry-shVglut2* along with AAVs expressing Cre in the thalamus of P0 wild-type mice (Figure S2Q). Analysis of barrels targeted by mCherry^+^ thalamocortical projections revealed no differences in the density of L4 PV^+^ interneurons between *shLacZ* and *shVglut1/2* mice at P12 (Figure S2R). We noticed that down-regulation of Slc17a6 expression in the thalamus (Figure S2S) was less effective than in the cortex (Figure S2N), so we used an alternative approach to manipulate thalamocortical activity. In brief, we performed unilateral infraorbital nerve (ION) sectioning at P9 and measured PV levels in cortical L4 at P12 (Figure S2T). We observed no differences in L4 PV levels between control and experimental animals (Figure S2U). These observations indicated that cortical activity, but not thalamic inputs, plays a more prominent role in controlling PV expression during a short developmental window near the onset of PV expression.

### PGC-1*α* mediates the effect of activity in PV^+^ interneurons

**Figure 2:**
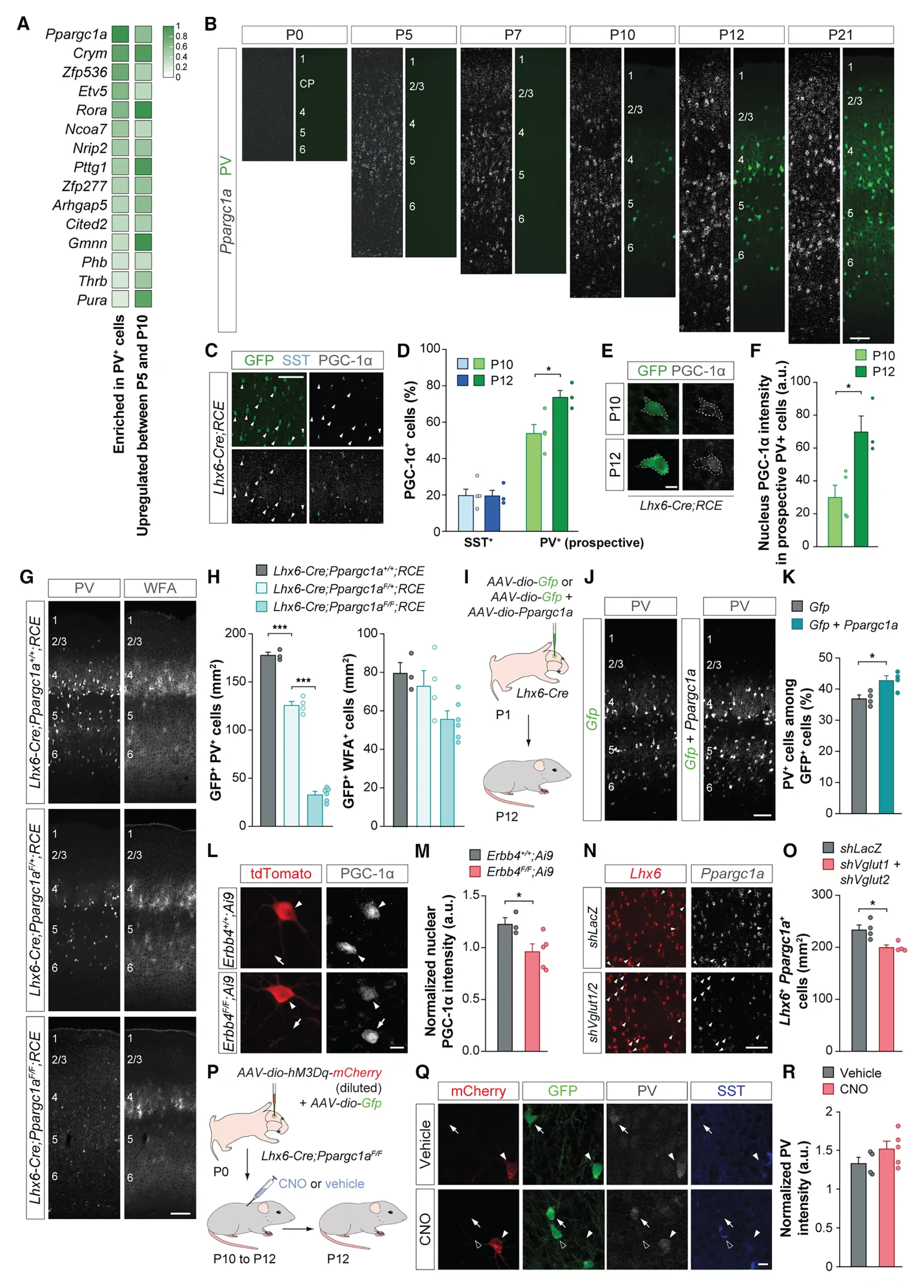
PV and WFA expression in prospective PV^+^ interneurons depends on the activity-dependent expression of PGC-1*α* (A) Selection criteria for genes encoding transcription factors or cofactors as candidates for mediating the activity-dependent maturation of PV^+^ interneurons. (B) PV and *Ppargc1a* expression in coronal sections through S1 at P0, P5, P7, P10, P12, and P21. (C) PGC-1*α* expression in SST^+^ cells and prospective PV^+^ interneurons in S1 of *Lhx6-Cre;RCE* mice at P12. Arrowheads point at prospective PV^+^ interneurons expressing PGC-1*α*. (D) Proportion of SST^+^ and prospective PV^+^ interneurons (recognized by the absence of SST expression) expressing PGC-1*α* at P10 and P12 in S1 of *Lhx6-Cre; RCE* mice. (E) PGC-1*α* expression in the nucleus of L2/3 MGE-derived cells in *Lhx6-Cre;RCE* mice at P10 and P12. (F) PGC-1*α* levels in the nucleus of prospective L2/3 PV^+^ interneurons at P10 and P12 in S1 of *Lhx6-Cre;RCE* mice. (G) PV and WFA expression in coronal sections through S1 from control, conditional heterozygous, and conditional homozygous *Ppargc1a* mutant mice at P12. (H) Density of GFP^+^ PV^+^ cells and GFP^+^ WFA^+^ cells across all layers in S1 of control, conditional heterozygous, and conditional homozygous *Ppargc1a* mice at P12. (I) Experimental design. (J) GFP and PV expression in coronal sections through S1 from *Gfp* and *Gfp* + *Ppargc1a* injected mice at P12. (K) Proportion of GFP^+^ infected cells expressing PV across all layers in S1 of *Gfp* control and *Ppargc1a* overexpression mice at P12. (L) PGC-1*α* expression in *E2-Cre*-infected and uninfected L5 PV^+^ cells from *ErbB4^+/+^;Ai9* and *ErbB4^F/F^;Ai9* mice at P12. Arrows and arrowheads point at un-infected and infected cells, respectively. (M) Nuclear PGC-1*α* levels in infected L5 PV^+^ cells normalized by nuclear PGC-1*α* levels in neighboring uninfected L5 PV^+^ cells. (N) *Ppargc1a* expression in *Lhx6*^+^ cells from control (*shLacZ*) and experimental (*shVglut1 + shVglut2*) mice at P12. Arrowheads point at *Lhx6*^+^ *Ppargc1a*^–^ cells. (O) Density of *Lhx6*^+^ *Ppargc1a*^+^ cells across all layers in S1 from control (*shLacZ*) and experimental (*shVglut1 + shVglut2*) mice at P12. (P) Experimental design. (Q) PV expression in prospective L2/3 PV^+^ cells (recognized by the absence of SST expression) expressing mCherry or GFP in control (vehicle) and experimental (CNO) conditions at P12 in *Lhx6-Cre; Ppargc1a^F/F^* mice. Arrows point at GFP^+^ PV^+^ cells, arrowheads at mCherry^+^ PV^+^ cells, and open arrowheads at a GFP^+^ SST^+^ cell. (R) PV levels in S1 L2/3 mCherry^+^ prospective PV^+^ cells normalized by PV levels in neighboring mCherry^–^ GFP^+^ prospective PV^+^ cells at P12. Data are presented as mean ± SEM. Scale bars, 10 μm (E, L, and Q) and 100 μm (B, C, G, J, and N). *p < 0.05, ***p < 0.001. See Table S5 for details on statistics. Related to Figures S1 and S3.

We hypothesized that transcriptional modulators might be responsible for driving a broad program of terminal differentiation in PV^+^ interneurons in response to an increase in neuronal activity. To identify such factor(s), we mined interneuron RNA sequencing (RNA-seq) datasets from the early postnatal mouse cortex^59^ and screened for transcriptional modulators enriched in PV^+^ interneurons whose expression increased between P5 and P10. We identified *Ppargc1a*, which encodes the transcriptional modulator PGC-1*α*, as the leading candidate on this list (Figure 2A).

We investigated whether the expression of *Ppargc1a* precedes the onset of PV expression in the postnatal cortex. *Ppargc1a* begins to be expressed in the neocortex around P5, and from P7 onward—before the onset of PV expression and perineuronal net formation—it concentrates in sparse somas spanning L2 to L6 (Figures 2B, S1A, and S1B). Most cortical cells containing high *Ppargc1a* mRNA levels are MGE-derived interneurons (Figures S3A and S3B), and the proportion of MGE-derived interneurons expressing *Ppargc1a* increases progressively until reaching a plateau at P12 (Figures S3A and S3C). We also analyzed the proportion of MGE-derived cells expressing PGC-1*α* during the onset of PV expression in the neocortex by breeding *Lhx6-Cre* mice with *RCL^Gfp/+^* (RCE) reporter mice.

Most MGE-derived cells expressing PGC-1*α* are prospective PV^+^ interneurons (GFP^+^ SST^-^ cells), and their proportion increases between P10 and P12 (Figures 2C and 2D). PGC-1*α* concentrates in the nucleus of prospective PV^+^ interneurons during this period (Figures 2E and 2F). By contrast, the proportion of SST^+^ cells expressing PGC-1*α* remains relatively low (Figures 2C and 2D).

To investigate whether PGC-1*α* is necessary for the expression of PV and WFA in prospective PV^+^ interneurons, we generated conditional mutants in which we deleted *Ppargc1a* specifically from MGE-derived cells. To this end, we bred *Lhx6-Cre* mice with mice carrying floxed *Ppargc1a* alleles and the RCE reporter to label Cre-expressing cells. We verified the effective loss of PGC-1*α* in *Lhx6-Cre;Ppargc1a^F/F^;RCE* mice (Figures S3D and S3E). The loss of PGC-1*α* does not impact the density or laminar distribution of MGE-derived interneurons (Figures S3F and S3G). However, mice lacking *Ppargc1a* exhibit a dose-dependent loss of PV expression, which is nearly absent in conditional homozygous *Ppargc1a* mutant mice (Figures 2G, 2H, and S3H). Conditional mutant mice also have fewer PV^+^ inter-neurons surrounded by perineuronal nets than controls, specifically in L4, where perineuronal nets are usually most abundant at P12 (Figures 2G, 2H, and S3H). By contrast, we found no difference in the density of SST^+^ interneurons in conditional homozygous *Ppargc1a* mutant mice compared with controls (Figures S3I–S3K). To establish whether PGC-1*α* is sufficient to accelerate the onset of PV expression in prospective PV^+^ interneurons, we injected S1 of P1 *Lhx6-Cre* pups with a cocktail of Cre-dependent AAVs expressing *Gfp* and *Ppargc1a* and analyzed the expression of PV at P12 (Figure 2I). PGC-1*α* over-expression increases the density of PV^+^ interneurons at this stage compared with controls (Figures 2J and 2K). These experiments demonstrate that PGC-1*α* is necessary and sufficient to drive the expression of PV and WFA in the early postnatal cortex.

We assessed whether PGC-1*α* mediates the role of activity in the onset of PV and WFA expression in PV^+^ interneurons. To this end, we examined whether reducing the density of excitatory inputs contacting infected PV^+^ interneurons (Figure 1D) or reducing glutamatergic neurotransmission in the neocortex (Figure 1G) impacts PGC-1*α* expression in prospective PV^+^ cells at P12. We found that both manipulations diminish nuclear PGC-1*α* levels in these cells and the number of *Lhx6*^+^ cells expressing *Ppargc1a* (Figures 2L–2O). Next, we increased the activity of prospective PV^+^ cells between P10 and P12 using activating DREADDs in *Lhx6-Cre;Ppargc1a^F/F^* mice (Figure 2P). In contrast to the results in wild-type mice (Figures 1B and 1C), we found no differences in PV and WFA expression between vehicle- and CNO-injected mutant mice (Figures 2Q, 2R, and S3L). Conversely, the overexpression of *Ppargc1a* in *Lhx6-Cre* mice in both vehicle and CNO conditions (Figure S3M) is sufficient to rescue the impact of downregulating the activity of prospective PV^+^ cells between P10 and P12 (Figures S3N and S3O). These experiments revealed that PGC-1*α* mediates the effect of neuronal activity on PV and WFA expression in PV^+^ interneurons during early postnatal development.

### PGC-1*α* controls the terminal differentiation of PV^+^ interneurons

**Figure 3:**
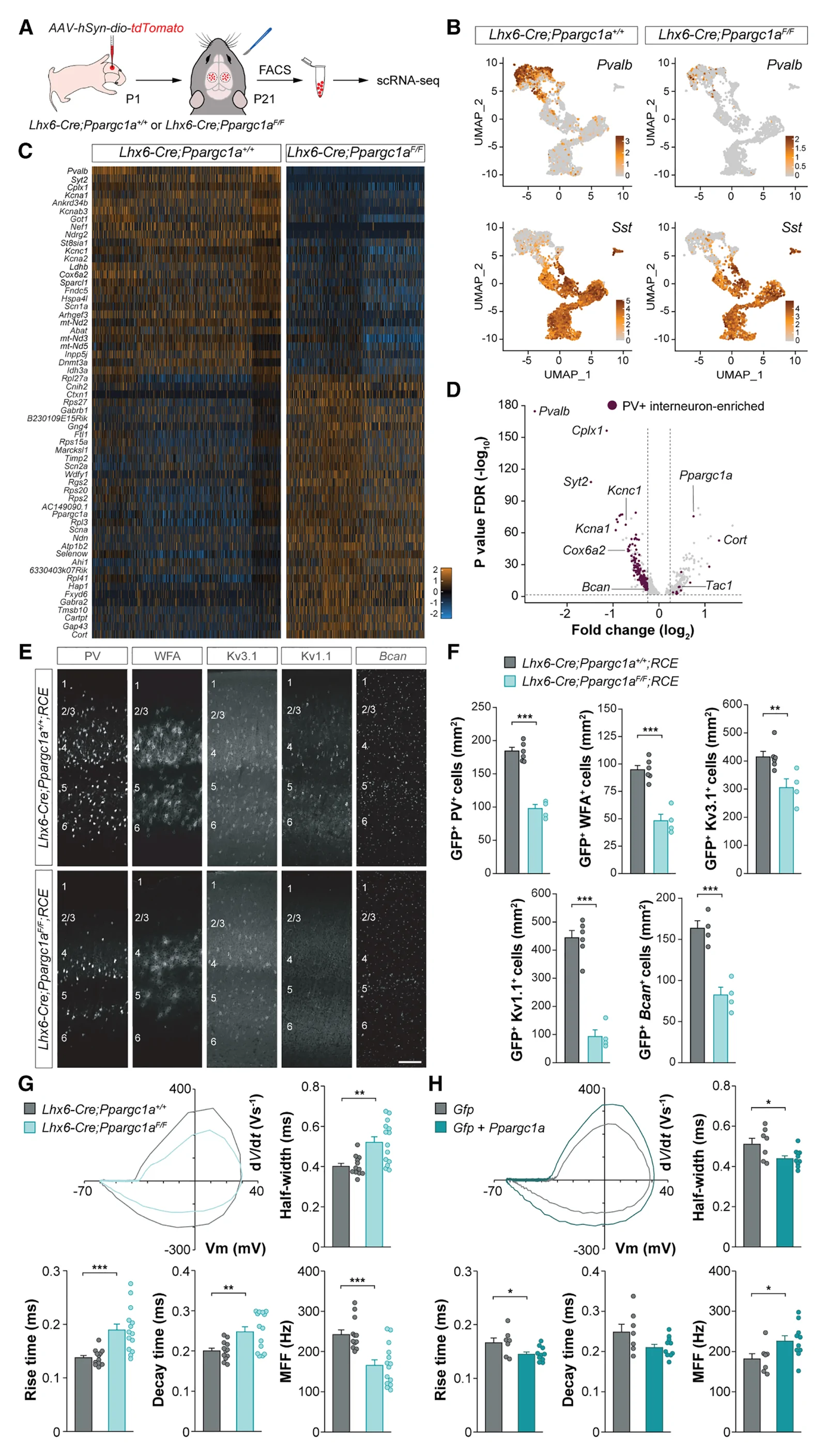
PGC-1*α* is a master regulator of the maturation of cortical PV+ interneurons (A) Experimental design. (B) Uniform manifold approximation and projection (UMAP) plots illustrating the expression of *Pvalb* and *Sst* in control and conditional homozygous *Ppargc1a* mice at P21. (C) Heatmap representing DGE between PV^+^ interneurons in control and conditional homozygous *Ppargc1a* mice at P21. (D) Volcano plot illustrating differentially expressed genes (DEGs) between PV^+^ interneurons in control and conditional homozygous *Ppargc1a* mice at P21. A threshold at log_2_FC > ±0.25 was set. Genes enriched in PV^+^ interneurons are highlighted in magenta. (E) PV, WFA, Kv3.1, Kv1.1, and *Bcan* expression in coronal sections through S1 of control and conditional homozygous *Ppargc1a* mice at P21. (F) Density of GFP^+^ PV^+^, GFP^+^ WFA^+^, GFP^+^ Kv3.1^+^, GFP^+^ Kv1.1^+^, and GFP^+^ *Bcan*^+^ cells across all layers in S1 of control and conditional *Ppargc1a* mutant mice at P21. (G) Intrinsic properties of L2/3 PV^+^ interneurons in control and conditional *Ppargc1a* mutant mice at P21. Data points represent cells. MFF, maximum firing frequency. (H) Intrinsic properties of L2/3 PV^+^ interneurons in control and *Ppargc1a* overexpression mice at P21. Data points represent cells. MFF, maximum firing frequency. Data are presented as mean ± SEM. Scale bar, 100 μm (E). *p < 0.05, **p < 0.01, ***p < 0.001. See Table S5 for details on statistics. Related to Figures S4, S5, and S6.

Our previous experiments suggested that PGC-1*α* plays a critical role in the onset of PV and WFA expression, essential markers of mature PV^+^ interneurons.^10^ However, whether PGC-1*α* regulates a broader molecular program required for the terminal differentiation of cortical PV^+^ interneurons is unclear. To explore this possibility, we analyzed gene expression in MGE-derived interneurons from control (*Lhx6-Cre;Ppargc1a^+/+^*) and conditional *Ppargc1a* mutant (*Lhx6-Cre;Ppargc1a^F/F^*) mice using single-cell RNA sequencing (scRNA-seq). To this end, we injected Cre-dependent AAVs expressing tdTomato under the control of the neuron-specific human synapsin-1 gene promoter^60^ into S1 of P1 pups. We then generated single-cell suspensions from S1 at P21, isolated the labeled cells using fluorescence-activated cell sorting, and performed scRNA-seq (Figure 3A). We took this approach to avoid isolating endothelial cells, which are also recombined in *Lhx6-Cre* mice^61^ (Figures S3D and S3F).

Following quality control (Figure S4), we isolated 5,268 control and 3,139 mutant MGE-derived interneurons from three highly consistent experimental replicates for each genotype (Figures S5A and S5B). We merged the replicates, integrated control and mutant samples, and classified the cells into PV^+^ and SST^+^ identities using cluster assignment based on *Pvalb* and *Sst* gene expression (Figures 3B, S5C, and S5D). We verified this assignment using label transfer and found that *Ppargc1a* mutant PV^+^ and SST^+^ interneurons cluster with the corresponding control cells (Figures 3B and S5D). This suggests that PGC-1*α* does not control the fate of MGE-derived interneurons.

We performed differential gene expression (DGE) analysis and found that many genes highly characteristic of PV^+^ interneurons were downregulated in mutant PV^+^ interneurons compared with control neurons (Figure 3C). Because PV^+^ and SST^+^ have a common developmental origin and shared transcriptional programs,^5^ we identified PV-enriched genes by comparing the transcriptome of control PV^+^ and SST^+^ interneurons at P21 (Figure S5E) and analyzed their expression in mutant PV^+^ cells. We found that many PV-enriched genes were downregulated in PV^+^ interneurons lacking PGC-1*α*, including *Pvalb, Cplx1, Syt2, Kcnc1, Kcna1*, and *Bcan* (Figures 3D–3F; Table S2). These genes encode proteins controlling a broad range of critical properties in mature PV^+^ interneurons, comprising neurochemical (e.g., PV and brevican), electrophysiological (e.g., Kv3.1 and Kv1.1), and synaptic (e.g., complexin-1 and synaptotagmin-2) features. Comparatively fewer PV-enriched genes were upregulated in mutant PV^+^ interneurons than in controls (Figure 3D). We found *Ppargc1a* among the upregulated genes (transcripts lacking the floxed exons 3–5), suggesting compensatory effects for the lack of PGC-1*α*.

Many genes downregulated in conditional *Ppargc1a* mutants control critical electrophysiological properties of mature PV^+^ interneurons. Consistently, mutant PV^+^ interneurons exhibited slower AP kinetics, affecting AP rise time, decay time, and half-width, leading to a reduced maximum firing frequency at P21 (Figure 3G; Table S1). We also observed a higher input resistance and depolarized AP threshold, consistent with the relatively immature state of these cells.^28,30^ Conversely, *Ppargc1a* overexpression in PV^+^ interneurons during development drives mature properties, including faster AP kinetics and an increased maximum firing frequency at P21 (Figure 3H; Table S1). These results revealed that PGC-1*α* is necessary and sufficient for the functional maturation of PV^+^ interneurons.

Active repression seems necessary to avoid the expression of alternative fates in related developmental lineages.^62^ We wondered whether PV^+^ interneurons lacking PGC-1*α* express genes enriched in SST^+^ interneurons. We first identified SST-enriched genes by comparing the transcriptome of control PV^+^ and SST^+^ interneurons at P21 (Figure S5E) and analyzed their expression in mutant PV^+^ cells. Several SST-enriched genes were upregulated in PV^+^ cells lacking PGC-1*α* (Figure S6A; Table S2), including the gene encoding calbindin (*Calb1*), a calcium-binding protein frequently found in deep-layer SST^+^ interneurons^63^ (Figures S6A–S6C). Looking at gene expression changes more globally, we observed that PV-enriched genes were more frequently downregulated, and SST-enriched genes were more upregulated in mutant PV^+^ interneurons than control cells (Figure S6D). To investigate whether this pattern also applies to other cortical interneurons from more distant developmental lineages, we used a published dataset^64^ to identify genes enriched in those cells and examined their expression in PV^+^ interneurons lacking PGC-1*α*. Using this alternative approach, we verified that PV-enriched genes are significantly downregulated without PGC-1*α*. Some genes enriched in other interneuron sub-classes are differentially expressed in mutant PV^+^ interneurons, but only the expression of the SST-enriched gene set was significantly upregulated (Figure S6E).

**Figure 4:**
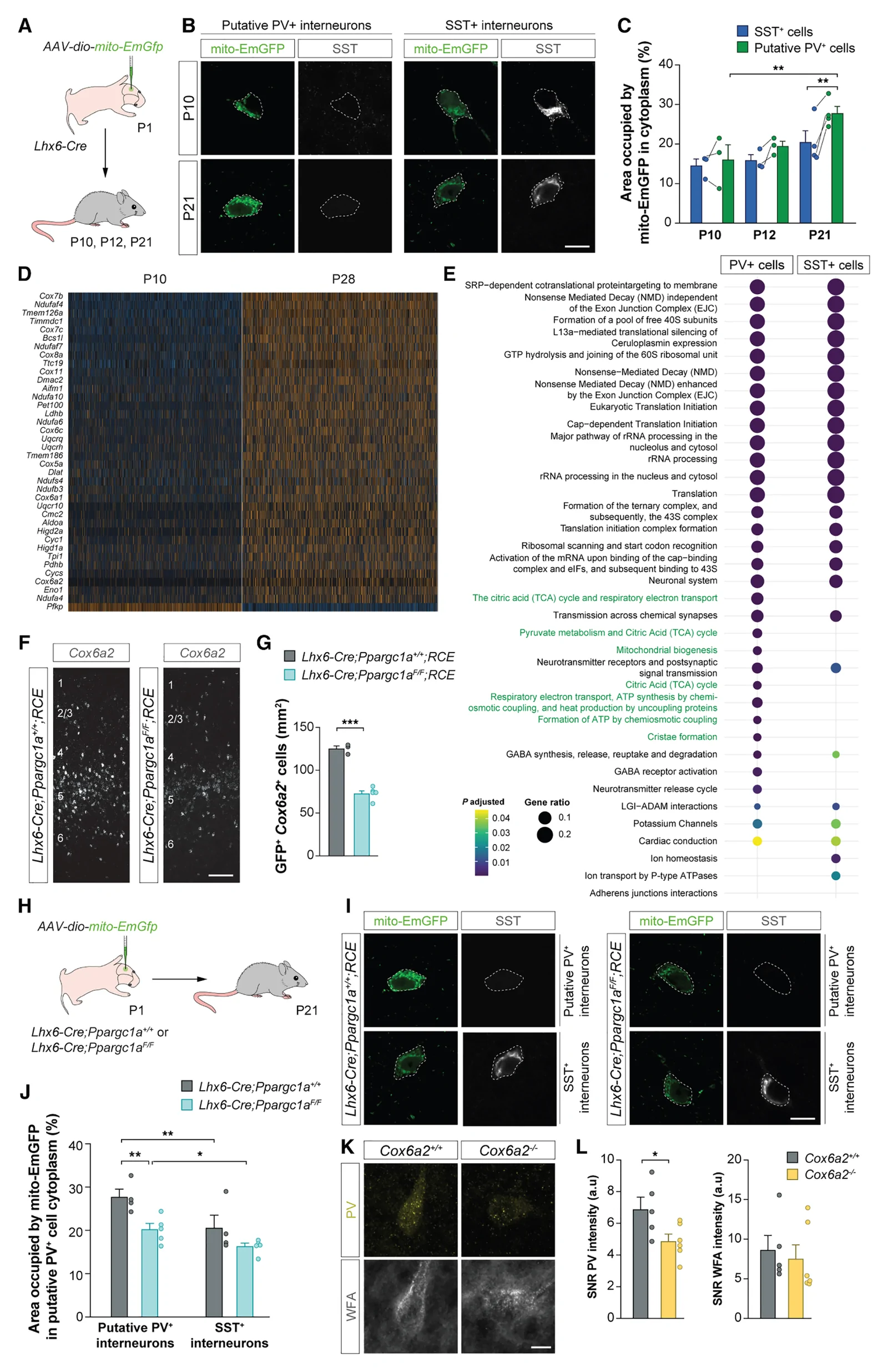
PGC-1*α* regulates mitochondrial function in cortical PV^+^ interneurons (A) Experimental design. (B) mito-EmGFP expression in the soma of PV^+^ interneurons (recognized by the absence of SST expression) and SST^+^ interneurons in L2/3 S1 of *Lhx6-Cre* mice at P10 and P21. (C) Area occupied by mito-EmGFP signal compared with the total cytoplasmic area in S1 L2–4 putative PV^+^ interneurons and SST^+^ cortical interneurons at P10, P12, and P21. (D) Heatmap representing differential expression of mitochondrial genes in PV^+^ interneurons between P10 and P28 (data extracted from Allaway and colleagues^69^). (E)Pathway enrichment analysis plot depicting the different cellular pathways affected by the absence of PGC-1*α*. Pathways associated with mitochondrial function are highlighted in green. (F) *Cox6a2* expression in coronal sections through S1 of control and conditional homozygous *Ppargc1a* mice at P21. (G) Density of GFP^+^ *Cox6a2*^+^ cells across all layers in S1 of control and conditional *Ppargc1a* mutant mice at P21. (H) Experimental design. (I) mito-EmGFP expression in the soma of PV^+^ and SST^+^ interneurons in L2/3 S1 of control and conditional *Ppargc1a* mutant mice at P21. PV^+^ interneurons are recognized by the absence of SST expression. (J) Area occupied by mito-EmGFP signal compared with the total cytoplasmic area in S1 L2–4 PV^+^ and SST^+^ cortical interneurons of control and conditional *Ppargc1a* mutant mice at P21. (K) PV and WFA expression in L4 S1 of control and *Cox6a2*^–/–^ mice at P12. (L) Signal-to-noise ratio (SNR) of PV and WFA intensities in L4 PV^+^ cells of S1 of control and *Cox6a2*^–/–^ mice at P12. Data are presented as mean ± SEM. Scale bars, 10 μm (B, I, and K) and 100 μm (F). *p < 0.05, **p < 0.01, ***p < 0.001. See Table S5 for details on statistics.

PV^+^ interneurons have considerable metabolic demands and contain considerably higher numbers of mitochondria than other interneurons and pyramidal cells.^65–67^ Using AAVs expressing a protein to tag mitochondria, mito-EmGFP^68^ (Figure 4A), we observed that putative PV^+^ and SST^+^ interneurons have similar mitochondrial content at P10 (Figures 4B and 4C). However, mitochondrial content increased disproportionately in PV^+^ interneurons compared with SST^+^ cells at P21 (Figures 4B and 4C). We also found that most mitochondrial genes are highly upregulated in PV^+^ interneurons during this period (Figure 4D). These experiments suggest that mitochondrial development in PV^+^ interneurons parallels the maturation of these cells.

Since PGC-1*α* is a master regulator of mitochondrial biogenesis in other cells,^70–72^ we wondered whether the loss of *Ppargc1a* may disrupt the metabolism of PV^+^ interneurons. Pathway enrichment analysis comparing DEGs in PV^+^ and SST^+^ interneurons revealed that terms associated with metabolic pathways are affected in mutant PV^+^ interneurons but not in mutant SST^+^ cells (Figure 4E). Many genes linked to mito-chondrial function and enriched in PV^+^ interneurons are downregulated in conditional *Ppargc1a* mutants (Figures 3C and 3D; Table S2), such as *Cox6a2*, a gene encoding a subunit of the cytochrome *c* oxidase that is highly specific to PV^+^ cells^73^ (Figures 4F and 4G). To examine whether the loss of PGC-1*α* affects the number of mitochondria in MGE-derived interneurons, we injected S1 of P1 control or *Ppargc1a* conditional mutants with AAVs expressing mito-EmGFP and analyzed mitochondrial content at P21 (Figure 4H). We found that the loss of *Ppargc1a* affects the number of mitochondria in PV^+^ interneurons but not in SST^+^ cells (Figures 4I and 4J). Since metabolism and mitochondria are drivers of maturation in many systems, including the brain,^68^ we also explored whether the loss of mitochondrial function impacts the maturation of PV^+^ interneurons at the onset of differentiation. To this end, we analyzed PV and WFA expression in control and *Cox6a2* mutant mice at P12. We found that PV intensity is reduced in *Cox6a2* mutants compared with controls (Figures 4K and 4L), indicating that mitochondrial dysfunction impacts the maturation of PV^+^ cells. Thus, PGC-1*α* is required to express an extensive molecular program that controls the terminal differentiation of PV^+^ interneurons and drives the biogenesis of the abundant mitochondria that characterize these cells. In addition to promoting the expression of many critical factors in these cells, this molecular program may involve repressing genes typically expressed by SST^+^ interneurons.

### PGC-1*α* directly regulates the expression of target genes in PV^+^ interneurons

Recent work suggests that mitochondrial metabolism sets the tempo of neuronal maturation,^68^ so disruption of mitochondrial biogenesis in PV^+^ interneurons lacking PGC-1*α* could slow down the terminal differentiation of these cells. Alternatively, PGC-1*α* may control the expression of target genes in PV^+^ interneurons by directly interacting with their promoters and enhancer sites. To begin distinguishing between these possibilities, we examined whether the maturation of PV^+^ interneurons is delayed or permanently disrupted by analyzing PV and WFA expression in control and conditional *Ppargc1a* mutants at P60. We found that loss of PGC-1*α* permanently impairs the maturation of PV^+^ cells across the cortex except for interneurons in L4, which have reached comparable levels to control mice by P60 (Figures S6F and S6G). These observations revealed that the maturation of many PV^+^ interneurons is not simply delayed in conditional *Ppargc1a* mutants, suggesting that PGC-1*α* may directly regulate the gene expression program of terminal differentiation in these cells.

**Figure 5:**
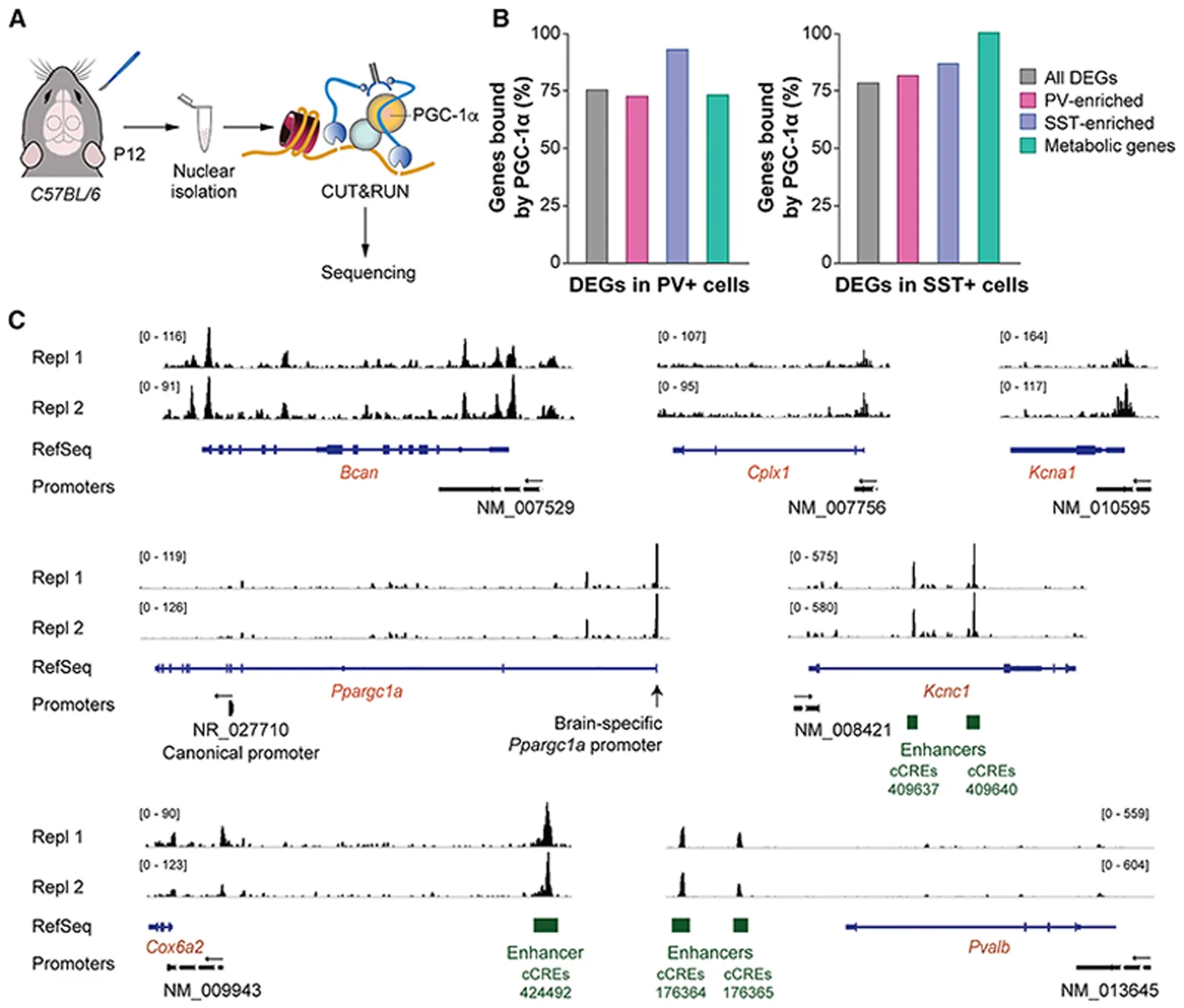
PGC-1*α* regulates the maturation of cortical PV^+^ interneurons through direct transcriptomic regulation (A) Experimental design. (B) DEGs between control and conditional homozygous *Ppargc1a* PV^+^ cells and SST^+^ cells, directly bound at their locus by PGC-1*α*. All DEGs (gray), PV-enriched genes (pink), SST-enriched genes (purple), and genes associated with metabolic function (green) were assessed. (C) Genome browser views of the genomic loci of the PV-enriched genes *Bcan, Cplx1, Kcna1, Ppargc1a, Kcnc1, Cox6a2*, and *Pvalb*. The black peaks represent the read coverage following PGC-1*α* CUT&RUN in two replicates at P12. Arrows indicate the direction of transcription. Related to Figure S7.

To assess this possibility, we dissected S1 from wild-type mice at P12, prepared nuclear suspensions, and conducted cleavage under targets and release using nuclease (CUT&RUN) assays using an antibody against PGC-1*α* (Figure 5A). We found that PGC-1*α* interacts at gene promoters or distal intergenic and intronic regions that may correspond to enhancer sites (Figure S7A). PGC-1*α* in cortical tissue is present at the locus of 75% of the genes whose expression is altered in PV^+^ cells lacking *Ppargc1a*, including PV- and SST-enriched markers and many metabolic genes (Figure 5B; Table S3). For instance, PGC-1*α* is present at the promoters of *Bcan, Cplx1*, and *Kcna1* (Figure 5C), genes highly enriched in PV^+^ interneurons and essential for acquiring their unique properties. PGC-1*α* is also present at enhancer sites^74^ for *Kcnc1* and potentially associated with *Pvalb* and *Cox6a2* (Figure 5C). In addition, PGC-1*α* seems to be able to modulate its expression in the neocortex by binding the *Ppargc1a* brain-specific promoter^75^ (Figure 5C). Remarkably, PGC-1*α* is associated with a significant fraction of genes encoding metabolic (e.g., *Cox8a, Ldhb*, and *Atp1b2*) and ribosomal (e.g., *Rpl30, Rpl41, Rps5*, and *Rps26*) proteins whose expression is dysregulated in mutant PV^+^ and SST^+^ interneurons (Figures 4E, S7B, and S7C; Table S3).

**Figure 6:**
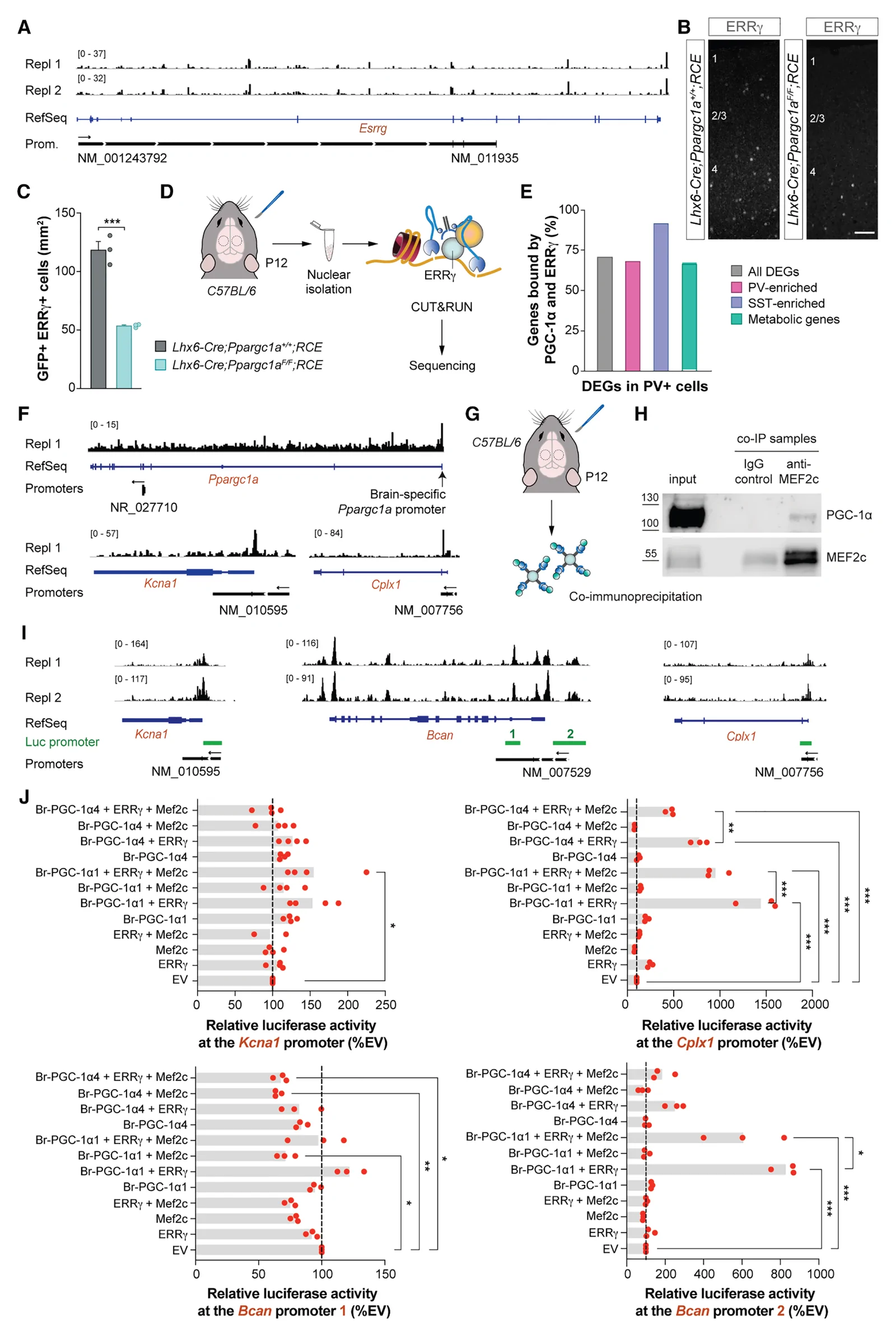
PGC-1*α*, ERRγ, and MEF2C directly regulate gene expression in PV^+^ interneurons (A) Genome browser view of the Esrrg genomic locus. The black peaks represent the read coverage following PGC-1*α* CUT&RUN in two replicates at P12. (B) ERRγ expression in coronal sections through S1 of control and conditional homozygous *Ppargc1a* mice at P21. (C) Density of GFP^+^ ERRγ^+^ cells across all layers in S1 of control and conditional *Ppargc1a* mutant mice at P21. (D) Experimental design. (E) DEGs between control and conditional homozygous *Ppargc1a* mutant PV^+^ cells, directly bound at their locus by PGC-1*α* and ERRγ. All DEGs (gray), PV-enriched genes (pink), SST-enriched genes (purple), and genes associated with metabolic function (green) were assessed. (F) Genome browser view of the genomic locus of *Ppargc1a, Kcna1*, and *Cplx1*. The black peaks represent the read coverage following ERRγ CUT&RUN at P12. (G) Experimental design. (H) Co-immunoprecipitation of MEF2C identifies the long isoform of PGC-1*α* as a binding partner. (I) Genome browser view of the genomic locus of *Kcna1, Bcan*, and *Cplx1*. The black peaks represent the read coverage following PGC-1*α* CUT&RUN in two replicates at P12. Gene promoters (in black), promoters chosen to conduct the luciferase assays (in green), and the size scale of the peaks are annotated. (J) Luciferase assay experiments using selected DNA sequences of *Kcna1, Cplx1*, and *Bcan* promoters bound by PGC-1*α*. Luciferase activity was assessed in the presence of different combinations of MEF2C, ERRγ, and the brain-specific short (PGC-1*α*4) and long (PGC-1*α*1) isoforms of PGC-1*α*. Data are presented as mean ± SEM. Scale bar, 100 μm (B). The arrows above the promoters indicate the direction of transcription. *p < 0.05, **p < 0.01, ***p < 0.001. See Table S5 for details on statistics. Related to Figure S8.

PGC-1*α* does not directly bind DNA but modulates gene expression by interacting with transcription factors and chromatin modifiers.^45,76^ For instance, members of the estrogen-related receptor (ERR) family are known coactivators of PGC-1*α* in several tissues.^77^ Among them, *Esrrg* mRNA is more abundantly and specifically expressed in PV^+^ interneurons than *Esrra* at P12 and P21 (Figures S8A, S8B, and S8E), and it extensively colocalizes with *Ppargc1a* (Figures S8C and S8F). We also observed that 75% of PV^+^ interneurons express ERRγ, while only 10% of SST^+^ interneurons do at P21 (Figures S8D and S8G). The specificity of ERRγ expression seems to be directly controlled by PGC-1*α* because it associates with the *Esrrg* locus in cortical cells at P12 (Figure 6A), and ERRγ levels are dramatically decreased in conditional *Ppargc1a* mutants (Figures 6B and 6C).

Although ERRα has been suggested to cooperate with PGC-1*α* to regulate *Pvalb* expression in the adult cortex,^78^ our previous observations indicated that ERRγ is the relevant coactivator of PGC-1*α* controlling the terminal differentiation of PV^+^ inter-neurons. To test this hypothesis, we dissected S1 from wild-type mice at P12, prepared nuclear suspensions, and conducted CUT&RUN assays using an antibody against ERRγ (Figure 6D). Approximately half of the loci (52.3%) bound by PGC-1*α* in cortical neurons are also bound by ERRγ. Like PGC-1*α*, ERRγ binds at gene promoters or distal intergenic and intronic regions (Figure S8H). We observed that 75% of the genes whose expression is altered in mutant PV^+^ interneurons are directly bound by ERRγ in cortical tissue, and most of these genes are also bound by PGC-1*α* (Figure 6E; Table S3). For example, ERRγ binds the promoters of *Kcna1, Cplx1*, and the brain-specific promoter of *Ppargc1a* (Figure 6F). These experiments revealed that PGC-1*α* and ERRγ cooperate in regulating the terminal differentiation of PV^+^ interneurons in the early postnatal neocortex.

We performed a motif enrichment analysis of PGC-1*α* binding sites to identify additional factors that may contribute to regulating PV-enriched genes. This analysis revealed that members of the myocyte enhancer factor 2 (Mef2) family are potential partners of PGC-1*α* in the neocortex (Figure S7D). Of note, Mef2c has been previously found to be involved in the differentiation of PV^+^ interneurons^79,80^ and is expressed by most MGE-derived interneurons at P12 (Figures S8J–S8L). We discovered that Mef2c interacts with the long isoform of PGC-1*α* (PGC-1*α*1) at P12 (Figures 6G and 6H), even though the short isoform (PGC-1*α*4) is much more abundant in the neocortex at this age (Figure S8M).

Since Mef2c is already expressed in cortical neurons at embryonic stages,^79,80^ we wondered whether this transcription factor may regulate the expression of PGC-1*α* in developing PV^+^ interneurons. To this end, we bred *Lhx6-Cre* mice with mice carrying floxed *Mef2c* alleles and with *Ai9* reporter mice to label Cre-expressing cells. In contrast to conditional *Ppargc1a* mutants, we found that the loss of Mef2c decreases the density and alters the laminar distribution of MGE-derived interneurons (Figures S8N and S8O), suggesting that Mef2c plays a role in the generation, migration, and/or survival of PV^+^ interneurons. Nevertheless, we found that, within the surviving MGE-derived cells, mice lacking one or two *Mef2c* alleles exhibit a dose-dependent loss of PGC-1*α* in MGE-derived interneurons, which is accompanied by a parallel reduction in PV levels (Figures S8N and S8O).

The previous experiments suggested that Mef2c may be required to express *Ppargc1a* and may subsequently form a complex with PGC-1*α* to regulate the terminal differentiation of PV^+^ interneurons in response to neuronal activity in the postnatal cerebral cortex. Motif enrichment analysis at the loci bound by ERRγ also showed Mef2c as a potential interactor (Figure S8I). To decipher how PGC-1*α*, ERRγ, and Mef2c may regulate the expression of PV-enriched genes, we performed luciferase experiments in vitro with the long (α1) and short (α4) brain isoforms of PGC-1*α*.^81^ We assessed whether different combinations of PGC-1*α*, ERRγ, and Mef2c regulate the expression of *Kcna1, Bcan*, and *Cplx1* by binding at the promoter of these genes on specific DNA sequences identified by the PGC-1*α* CUT&RUN experiments (Table S4). We chose to test these targets as they each regulate different properties of mature PV^+^ interneurons. We found that PGC-1*α*1, ERRγ, and Mef2c promote the expression of *Kcna1*. Both PGC-1*α* isoforms (with the long isoform consistently having the most vigorous effect) and ERRγ can promote the expression of *Cplx1* and *Bcan*, but only when binding to *Bcan* promoter 2 (Figure 6J). This is consistent with previous work suggesting that ERRγ can directly interact with the two isoforms of PGC-1*α*, whereas only the long isoform of PGC-1*α* has a Mef2c binding site.^82^ Interestingly, Mef2c can negatively regulate *Bcan* expression when binding at *Bcan* promoter 1, suggesting that PGC-1*α*, ERRγ, and Mef2c do not always work cooperatively (Figure 6J). Similarly, Mef2c reduces the strong activation of *Cplx1* expression by ERRγ and PGC-1*α*1 or PGC-1*α*4, but it does not inhibit *Cplx1* expression alone or with PGC-1*α*. These results demonstrate that PGC-1*α*, through its interaction with ERRγ, directly controls the expression of genes required for the maturation of PV^+^ interneurons.

### PGC-1*α* is required for PV^+^ interneuron subtype specification

**Figure 7:**
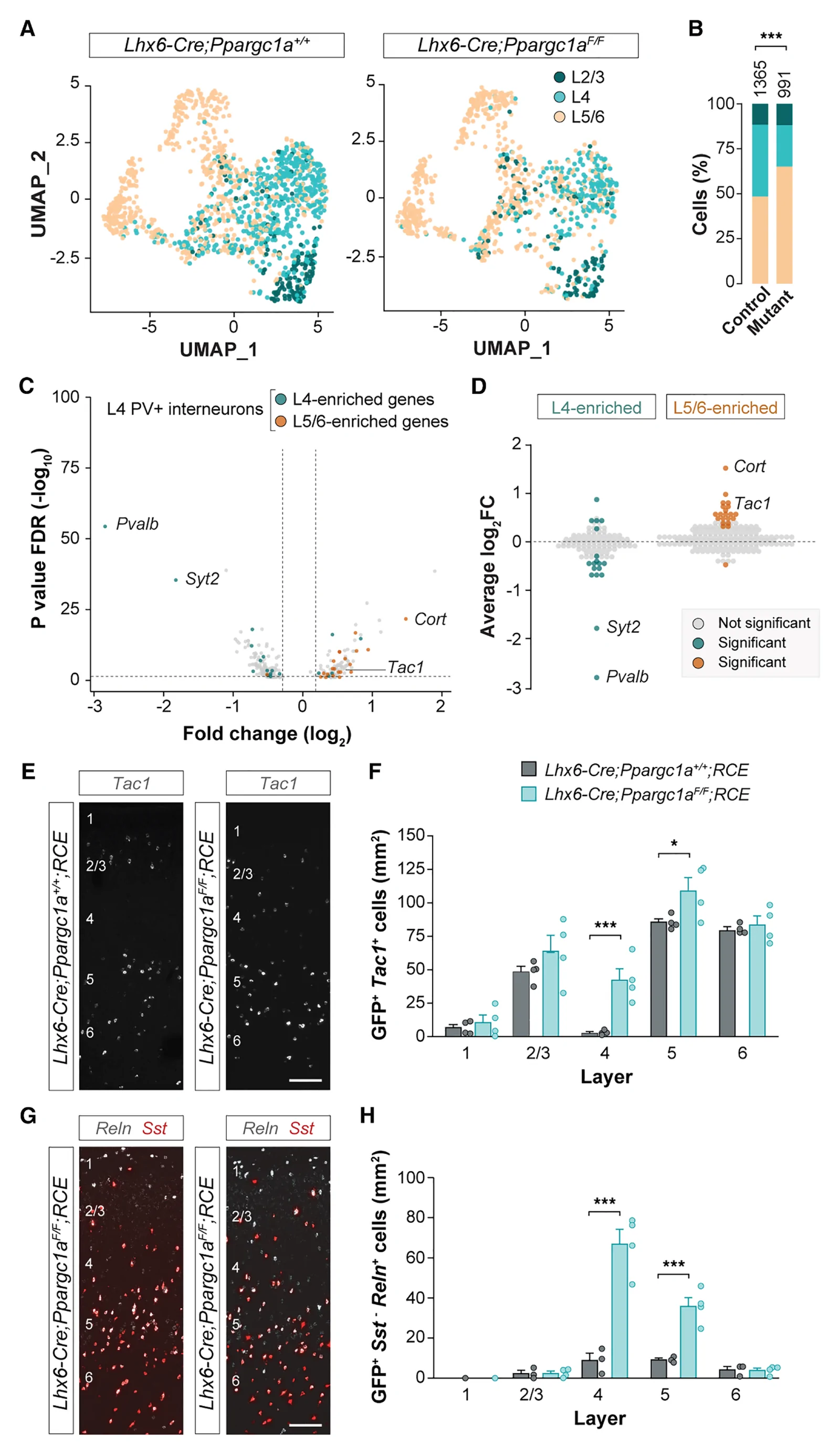
PGC-1*α* regulates subtype specification in PV^+^ interneurons (A) UMAP plots illustrating the assignment of L2/3, L4, and L5/6 subtypes of PV^+^ interneurons in control and conditional homozygous *Ppargc1a* mice at P21. (B) Proportion of PV^+^ interneurons identified as L2/3, L4, and L5/6 subtypes in control and conditional homozygous *Ppargc1a* mice at P21. (C) DEGs between L4 PV^+^ interneurons in control and conditional homozygous *Ppargc1a* mice at P21. A threshold at log_2_FC > ± 0.25 was set. Genes enriched in L4 and L5/6 PV^+^ interneurons are highlighted in cyan and orange, respectively. (D) Expression changes (average log_2_FC) in L4 PV^+^ interneurons between control and conditional homozygous *Ppargc1a* mice at P21 for genes enriched in L4 or L5/6 PV^+^ interneurons. A threshold at log_2_FC > ± 0.25 was set. Significant changes in expression are highlighted in cyan and orange for genes enriched in L4 and L5/6 PV^+^ interneurons, respectively. (E) *Tac1* expression in coronal sections through S1 of control and conditional homozygous *Ppargc1a* mice at P21. (F) Density of GFP^+^ *Tac1*^+^ cells across all cortical layers of S1 in control and conditional *Ppargc1a* mutant mice at P21. (G) *Reln* expression in PV^+^ interneurons (recognized by the absence of *Sst* expression) in coronal sections through S1 of control and conditional *Ppargc1a* mutant mice at P21. (H) Density of GFP^+^ *Sst*^-^ *Reln*^+^ cells across all S1 layers in control and conditional *Ppargc1a* mutant mice at P21. Data are presented as mean ± SEM. Scale bars, 100 μm (E and G). *p < 0.05, ***p < 0.001. See Table S5 for details on statistics.

Several subtypes of PV^+^ interneurons segregate across different layers of the cortex. Although they share many features, each PV^+^ interneuron subtype has specific molecular, electrophysiological, and morphological characteristics.^83^ It has been proposed that neuronal activity may instruct the identity of cortical interneurons,^4^ but this hypothesis has not been validated. Since *Ppargc1a* regulates the maturation of PV^+^ interneurons in an activity-dependent manner, we explored whether this gene may also contribute to the diversification of PV^+^ cells into different subtypes. To this end, we performed label transfer from a reference adult cortical interneuron dataset^83^ to identify layer-specific subtypes of PV^+^ interneurons in our scRNA-seq dataset (Figure 7A). We found that the lack of PGC-1*α* caused a shift in the proportion of PV^+^ interneuron subtypes, as defined by their transcriptional features, with an overrepresentation of L5/6 PV^+^ cells at the expense of L4 PV^+^ interneurons (Figure 7B). Although L4 PV^+^ interneurons can overcome the lack of PGC-1*α* over time and acquire the general properties of PV^+^ interneurons, this observation suggests that some of these cells may do so at the expense of their specific L4 identity.

To investigate whether the remaining mutant L4 PV^+^ cells were abnormally specified, we performed a DGE analysis between L4 and L5/6 control PV^+^ interneurons at P21 and examined the expression of these genes in L4 PV^+^ interneurons lacking PGC-1*α* (Figures 7C and 7D). We found that genes characteristic of L5/6 PV^+^ interneurons, such as *Tac1* and *Cort*, are abnormally upregulated in L4 PV^+^ interneurons in conditional *Ppargc1a* mutants (Figures 7C–7F). Since *Tac1* and *Cort* are also expressed in L2/3 PV^+^ interneurons, we also examined the expression of *Reln*, enriched in L5 PV^+^ interneurons.^83^ We found that *Reln* is also abnormally upregulated in mutant L4 PV^+^ interneu-5568 Cell *188*, 5555–5575, October 2, 2025 rons (Figures 7G and 7H). Thus, PGC-1*α* is required in these cells to acquire properties associated with their terminal differentiation. Altogether, these observations suggest that PGC-1*α* is not only required for the terminal differentiation of PV^+^ interneurons but also seems to contribute to their subtype diversification in the early postnatal cortex.

## Discussion

The mammalian cerebral cortex exhibits a characteristic protracted development. Cortical neurons are specified at embryonic stages but only acquire their adult features at relatively advanced stages of postnatal development. Here, we identify the cellular and molecular mechanisms controlling the terminal differentiation of PV^+^ interneurons, a major group of GABAergic cells in the cerebral cortex.

### Activity-dependent maturation of cortical PV^+^ interneurons

Our experiments revealed that the maturation of PV^+^ interneurons critically depends on neuronal activity. Changes in neuronal activity directly impact the onset of PV expression and perineuronal net formation, and crucially, so does PGC-1*α* expression, which we identified as a master regulator of the terminal differentiation of PV^+^ interneurons. PV^+^ interneurons receive transient inhibitory connections from SST^+^ interneurons during the first week of postnatal development,^56,84,85^ which effectively prevent their maturation during this period.^24^ The number and strength of glutamatergic inputs received by PV^+^ interneurons increase progressively during the second week of postnatal development,^28,30,84^ and this shift in balance between excitation and inhibition in PV^+^ interneurons seems to trigger the terminal differentiation of these cells. Our experiments suggest that local cortical activity is more critical in this process than thalamocortical inputs. This contrasts with previous work indicating that sensory experience influences the development of PV^+^ interneurons in the somatosensory and visual cortices.^33,86^ It should be noted, however, that our experiments only explored a limited critical window close to the onset of PV expression and that downregulation of *Vglut2* in the thalamus was less effective than in the cortex.

PGC-1*α* controls the expression of a PV-specific transcriptional program. However, the precise molecular mechanisms leading to the onset of PGC-1*α* expression in PV^+^ interneurons in response to activity remain unknown. Experiments in neuronal cultures indicated that PGC-1*α* expression is modulated by Ca^2+^, AMPK (AMP-activated protein kinase), and p38MAPK (mitogen-activated protein kinase) signaling in response to changes in activity, likely by recruiting the cAMP response element-binding protein (CREB) to the *Ppargc1a* locus.^87–89^ The expression of MEF2 transcription factors is also promoted by glutamatergic signaling in neuronal cultures.^90^ Interestingly, MEF2 transcription factors cooperate with CREB to regulate the expression of PGC-1*α* in skeletal muscle,^91^ and our experiments indicate that Mef2c is required for the expression of PGC-1*α* in developing PV^+^ interneurons. However, Mef2c is expressed by putative PV^+^ interneurons long before *Ppargc1a* is expressed in these cells.^69^ This suggests that additional mechanisms contribute to the expression of PGC-1*α* in PV^+^ interneurons. For instance, neuronal activity could promote activating post-translational modifications on Mef2c^92^ or induce epigenetic changes regulating PGC-1*α* expression, as shown in other tissues.^93–95^

### PGC-1*α* translates activity signals into specific transcriptional programs

Our experiments revealed that PGC-1*α* is required to mediate the effect of neuronal activity in the maturation of PV^+^ interneurons. In other organs, PGC-1*α* coordinates cellular responses to environmental cues.^40,41^ For instance, PGC-1*α* expression increases in response to cold in skeletal muscle and brown fat adipose tissue for adaptive thermogenesis,^70,96^ whereas in the liver, it is induced by fasting to promote gluconeogenesis.^71,97,98^ In the brain, PGC-1*α* has been co-opted by cortical PV^+^ interneurons to link their terminal differentiation to the increase in neuronal activity that characterizes the second postnatal week of postnatal development.

PGC-1*α* function in skeletal muscle, brown fat, and liver has been primarily linked to mitochondrial biogenesis and metabolism.^70,99,100^ Analysis of mutant mice revealed that PGC-1*α* is dispensable for mitochondrial biogenesis per se, but it is required to express many mitochondrial genes in response to environmental cues.^71,101^ We observed that PGC-1*α* is also dispensable for basal mitochondrial biogenesis in PV^+^ and SST^+^ interneurons. However, it is critically required for the surge in mitochondria that characterizes adult PV^+^ interneurons^65–67^ and for the expression of specific mitochondrial genes in these cells. PGC-1*α* could trigger the maturation of PV^+^ interneurons by promoting mitochondrial biogenesis and oxidative phosphorylation.^68^ Consistently, conditional deletion of the cytochrome *c* subunit Cox6a2 from PV^+^ interneurons alters the onset of PV expression (this study) and reduces PV levels and perineuronal nets in the adult cortex.^73,102^ However, these defects do not match the severity of phenotypes observed in conditional *Ppargc1a* mutant mice. Moreover, PGC-1*α* directly regulates the transcription of non-mitochondrial genes in PV^+^ interneurons, including voltage-dependent channels critical for their adult electrophysiological properties, revealing critical functions besides mitochondrial biogenesis. Consistently, PGC-1*α* regulates the expression of cell-type-specific programs in skeletal muscle, promoting the differentiation of slow-twitch muscle fibers.^103^ Thus, PGC-1*α* functions in the brain as a master regulator of PV^+^ interneurons, promoting mitochondrial biogenesis and cell-type-specific transcriptional programs driving their terminal differentiation.

PGC-1*α* interacts with ERRγ, whose expression is also directly controlled by PGC-1*α*, to regulate the expression of PV-enriched genes. The long isoform of PGC-1*α* seems critical for this function, although its short isoform is more highly expressed in S1 at P12. It is conceivable that long and short isoforms of PGC-1*α* regulate different aspects of the maturation of PV^+^ interneurons because only the short isoform can enter the mitochondria and interact with mitochondrial DNA.^100^ It has been previously suggested that ERRα, another member of the ERR family, is involved in the maturation of PV^+^ interneurons.^78^ However, ERRα is expressed at relatively low levels in the cerebral cortex and is not specific to PV^+^ interneurons, suggesting that ERRγ is the preferred coactivator of PGC-1*α* in these cells.

In addition to promoting the differentiation of PV^+^ interneurons through the expression of PV-specific genes, PGC-1*α* represses the expression of SST-specific genes in these cells. This observation suggests that despite the early specification of PV^+^ and SST^+^ interneurons,^79,104,105^ additional mechanisms contribute to consolidating their respective identities in the postnatal cortex. Consistent with this idea, we found that PGC-1*α* modulates key features of L4 PV^+^ interneurons. In sum, activity-dependent mechanisms mediated by PGC-1*α* are not only required for the terminal differentiation of PV^+^ interneurons but also contribute to the expression of subtype-specific transcriptional programs.

### Implications for neurodevelopmental disorders

The maturation of fast somatic inhibition by PV^+^ interneurons is critical for the emergence of sparse activity in cortical networks,^24,106,107^ the regulation of critical periods of plasticity,^25–27^ and the generation of gamma oscillations essential for information processing.^14,15,108^ Unsurprisingly, dysfunction of cortical PV^+^ interneurons has been repeatedly linked to neurodevelopmental disorders.^109,110^ Postmortem studies have shown that PV expression in the cerebral cortex is consistently downregulated in autism spectrum disorder (ASD) and schizophrenia,^111–115^ and mice carrying loss-of-function variants of developmental genes associated with these conditions exhibit reduced PV levels in the cerebral cortex.^116–120^ Although mutations in the *Ppargc1a* locus have not been directly linked to neurodevelopmental disorders, loss-of-function mutations in *MEF2C* are linked to intellectual disability, ASD, and schizophrenia.^121^ In addition, several genes whose variation has been linked to neurodevelopmental disorders regulate the development of excitatory synapses on PV^+^ interneurons,^48,122,123^ and these early wiring defects may impair PGC-1*α* expression and the maturation of these cells. PV^+^ interneurons also seem more susceptible to redox dysregulation and oxidative stress than other neurons.^124^ Thus, defects in the maturation of PV^+^ interneurons may render these cells unable to cope with subsequent environmental insults, leading to cortical dysfunction and disease.

### Limitations of the study

Our experiments revealed that the maturation of PV^+^ interneurons depends on neuronal activity and is mediated by the expression and transcriptional function of PGC-1*α*. Changes in neural activity over a short period influence the onset of PV and WFA expression in PV^+^ cells, but the intrinsic electro-physiological properties of these cells take longer to mature. The requirement of PGC-1*α* in mediating the role of activity in this context and the fact that the loss of PGC-1*α* alters the intrinsic electrophysiological properties of PV^+^ interneurons by P21 nevertheless suggest that neural activity and PGC-1*α* are interlinked in mediating the functional maturation of these cells. The activity-dependent signaling cascade leading to the expression and localization of PGC-1*α* to the nucleus of PV^+^ interneurons remains to be characterized. In addition, some defects observed in PV^+^ interneurons lacking PGC-1*α*, especially in L4, are partially compensated over time, suggesting that other molecular programs may contribute to the maturation of these cells. PGC-1*α* is also expressed in approximately 20% of SST^+^ interneurons, but its role in these cells remains unclear. Finally, we did not explore sex-dependent differences in the maturation of cortical PV^+^ interneurons. This may be particularly relevant in the context of neurodevelopmental disorders.

## Resource Availability

### Lead contact

Further information and requests for resources and reagents should be directed to the lead contact, Oscar Marín (oscar.marin@kcl.ac.uk).

### Materials availability

- Plasmids used to generate viruses in this study will be deposited on Addgene.

## Star★Methods

### Key Resources Table

**Table:** 

REAGENT or RESOURCE	SOURCE	IDENTIFIER
Antibodies		
Chicken anti-GFP (1:1000)	Aves Labs	Cat #GFP-1020; RRID: AB_10000240
Donkey anti-Chicken, Alexa Fluor 488 (1:200)	Jackson ImmunoResearch	Cat #703-545-155; RRID: AB_2340375
Donkey-anti-Guinea pig, Alexa Fluor 647 (1:250)	Jackson ImmunoResearch	Cat #706-605-148; RRID: AB_2340476
Donkey anti-Goat, Alexa Fluor 555 (1:400)	ThermoFisher	Cat #A21432; RRID: AB_2535853
Donkey anti-Mouse, Alexa Fluor 647 (1:400)	ThermoFisher	Cat #A31571; RRID: AB_162542
Donkey anti-Rabbit, Alexa Fluor 488 (1:400)	ThermoFisher	Cat #A21206; RRID: AB_2535792
Donkey anti-Rabbit, Alexa Fluor 555 (1:400)	ThermoFisher	Cat #A31572; RRID: AB_162543
Donkey anti-Rabbit, Alexa Fluor 647 (1:400)	ThermoFisher	Cat #A31573; RRID: AB_2536183
Donkey anti-Rabbit, Alexa Fluor 405 (1:200)	Abcam	Cat #Ab175651; RRID: AB_2923541
Guinea pig anti-Parvalbumin (1:1000)	Synaptic Systems	Cat #195-004; RRID: AB_2156476
Goat anti-Guinea pig, Alexa Fluor 488	Thermo Fisher	Cat #A11073; RRID: AB_2534117
Goat anti-mCherry (1:500)	Antibodies Online	Cat #ABIN1440057; RRID: AB_11208222
Goat anti-Mouse IgG1, Alexa Fluor 647	ThermoFisher	Cat #A21240; RRID: AB_2535809
Goat anti-Mouse IgG2a, Alexa Fluor 488	ThermoFisher	Cat #A21131; RRID: AB_2535771
Goat anti-Mouse IgG2a, Alexa Fluor 555	ThermoFisher	Cat #A21137; RRID: AB_2535776
Goat anti-Mouse IgG2a, Alexa Fluor 647	ThermoFisher	Cat #A21241; RRID: AB_2535810
Mouse anti-ERRγ (1:100 IHC, 2 μg CUT&RUN)	R&D Systems	Cat #PP-H6812-00; RRID: AB_2100280
Mouse anti-Kv1.1 (1:1000)	Antibodies Incorporated	Cat #75-105; RRID: AB_2128566
Mouse anti-Kv3.1 (1:1000)	Antibodies Incorporated	Cat #75-041; RRID: AB_2131480
Mouse anti-PGC-1*α* (1:200)	Santa Cruz	Cat #sc-518025; RRID: AB_2890187
Mouse anti-PGC-1*α* (2 μg CUT&RUN)	Millipore	Cat #ST1202; RRID: AB_2237237
Rabbit anti-dsRed (1:500)	Clontech	Cat #632496; RRID: AB_10013483
Rabbit anti-Fos (1:500)	Cell Signaling	Cat #2250; RRID: AB_2247211
Rabbit anti-Mef2c (1:100 IHC, 8 μg co-IP, 1:500 WB)	ProteinTech	Cat #18290-1-AP; RRID: AB_2142849
Rabbit anti-Mouse IgG	Abcam	Cat #ab6709; RRID: AB_956006
Rabbit anti-Parvalbumin (1:5000)	Swant	Cat #PV-27; RRID: AB_2631173
Rabbit anti-Somatostatin (1:3000)	BMA Biomedicals	Cat #T-4103
Rabbit IgG (8 ug co-IP)	Abcam	Cat #ab27478; RRID: AB_2616600
Streptavidin 555 (1:400)	ThermoFisher	Cat #S21381; RRID: AB_2307336
Streptavidin 647 (1:400)	Jackson ImmunoResearch	Cat #016-600-084; RRID: AB_2341101
Wisteria Floribunda Agglutinin	Sigma Aldrich	Cat #L-1516; RRID: AB_2620171
Critical commercial assays		
Mm-*Bcan*-O2-C2	ACDBio	Cat #531761-C2
*Mm-Cox6a2-C3*	ACDBio	Cat #461371-C3
*Mm-Esrra-O2-C2*	ACDBio	Cat #534441-C2
*Mm-Esrrg-C3*	ACDBio	Cat #495121-C3
*Mm-Lhx6-C2*	ACDBio	Cat #422791-C2
*Mm-Lhx6-C3*	ACDBio	Cat #422791-C3
*Mm-Ppargc1a-C1*	ACDBio	Cat #402121
*Mm-Reln-C2*	ACDBio	Cat #405981-C2
*Mm-Slc17a6-C2*	ACDBio	Cat #319171-C2
*Mm-Slc17a7-C1*	ACDBio	Cat #416631
Mm-*Sst*-C3	ACDBio	Cat #404631-C3
Mm-*7ac1*-C3	ACDBio	Cat #410351-C3
Papain Dissociation System	Worthington	Cat #LK003160
RNAscope Multiplex Fluorescent Assay v2	ACDBio	Cat #323110
Bacterial and virus strains		
pAAV-E2-Cre	A gift from Beatriz Rico	N/A
pAAV-hSyn-dio-GFP	A gift from Bryan Roth	Addgene #50457
pAAV-hSyn-dio-hM3D(Gq)-mCherry	Krashes et al.^125^	Addgene #44361
pAAV-hSyn-dio-hM4D(Gi)-mCherry	Krashes et al.^125^	Addgene #44362
pAAV-hSyn-frt-Dio-hM3D(Gq)-mCherry	Modol et al.^58^	Addgene#217398
pAAV-hSyn-dio-mito-EmGFP	This study (emGFP construct from Pierre Vanderhaeghen)	Addgene#240414
pAAV-hSyn-dio-shLacZ-mCherry	Favuzzi et al.^59^	N/A
pAAV-EF1a-dio-DSE-mCherry-PSE-shVglut1	This study	Addgene#240412
pAAV-EF1a-dio-DSE-mCherry-PSE-shVglut2	This study	Addgene#240413
pAAV-hSyn-EGFP-Cre	A gift from James M. Wilson	Addgene #105540
pAAV-hSyn-dio-tdTomato	A gift from Beatriz Rico	N/A
pAAV-hSyn-dio-Ppargc1a (long isoform)	This study	Addgene#240415
Deposited data		
scRNA-seq, CUT&RUN, analysis pipeline	This study	GEO: GSE256441, GSE256442, GSE256443
Images and data analysis	This study	KORDS:https://doi.org/10.18742/29198687
Experimental models: Organisms/strains		
Mouse: *Ai9* [B6.Cg-Gt(ROSA)26Sor^tm9(CAG-tdTomato)Hze^/J]	Madisen et al.^126^	JAX: 007909; RRID: IMSR_JAX:007909
Mouse: *Erbb4^F/F^* [Erbb4^tm1Fej^]	Golub et al.^127^	N/A
Mouse: *Lhx6-Fre* [B6;CBA-Tg(Lhx6-iCre)1Kess/J]	Fogarty et al.^61^	JAX: 026555; RRID: IMSR_JAX:026555
Mouse: *Mef2c^F/F^* [Mef2c^tm1Jjs^]	Vong et al.^128^	JAX: 025556; RRID: IMSR_JAX:025556
Mouse: *Nex^Cre/+^* [Neurod6^tm1(cre)Kan^]	Goebbels et al.^129^	N/A
Mouse: *Ppargc1a^F/F^* [Ppargc1a^tm2^-^1Brsp^]	Lin et al.^71^	JAX: 009666; RRID: IMSR_JAX:009666
Mouse: *RFE* [Gt(ROSA)26Sor^tm1^-^1(CAG-EGFP)Fsh^]	Sousa et al.^130^	JAX: 032037; RRID: MMRRC_032037-JAX
Mouse: *Sst^Cre/+^* [B6N.Cg-Ssttm2.1(cre)Zjh]	Taniguchi et al.^131^	JAX: 018973; RRID: IMSR_JAX:018973
Mouse: *SstFlp/+* [Ssttm3.1(Flpo)Zjh/J]	He et al.^132^	JAX: 028579; RRID:IMSR_JAX:02857 9
Mouse: *Fox6a2^-/-^* [Cox6a2^tm1Djg^]	Radford et al.^133^	RRID: MGI:3038743
Mouse: *G42* [CB6-Tg(Gad1-EGFP)G42ZjhJ]	Chattopadhyaya et al.^134^	JAX: 007677; RRID:IMRS_JAX:007677
Software and algorithms		
FIJI, ImageJ, Version 2.00-rc-69/1.52i	Open Software, ImageJ	RRID: SCR_002285, https://imagej.net/Fiji
MATLAB	MathWorks	RRID: SCR_001622, https://uk.mathworks.com/products/matlab.html
R Studio, Version 2023.06.2+561	R Studio team	RRID: SCR_000432, https://www.rstudio.com/
IGV, Version 2.16.0	IGV	RRID: SCR_011793, https://www.igv.org
Illustrator CC 2021	Adobe	RRID: SCR_010279, https://www.adobe.com/products/illustrator.html
Clampex 10.7	Molecular Devices	N/A
Clampfit 10.2	Molecular Devices	N/A

### Experimental Model and Study Participant Details

#### Mice

All experiments were performed following King’s College London Biological Service Unit’s guidelines and under license from the UK Home Office following the Animals (Scientific Procedures) Act 1986. Similar numbers of male and female mice were used in all experiments. Animals were maintained under standard laboratory conditions on a 12:12 light/dark cycle with water and food ad libitum. We used *Lhx6-Cre* mice [B6;CBA-Tg(Lhx6-icre)1Kess/J]^61^ to modulate the activity of PV^+^ interneurons at early postnatal stages (also crossed to *Sst^Flp/+^* mice [Sst^tm3.1(Flpo)Zjh/J^] in one set of experiments) and to overexpress the long isoform of *Ppargc1a* in PV^+^ interneurons. *ErbB4*-floxed mice [Erbb4^tm1Fej^]^127^ crossed to *Ai9* mice [Gt(ROSA)26Sor^tm9(CAG-tdTomato)Hze^]^126^ (JAX007909), and *Nex^Cre/+^* mice [Neurod6^tm1(cre)Kan^]^129^ were used to modulate the excitatory activity received by PV^+^ interneurons. ION sectioning experiments and thalamic *Vglut1/2* downregulation were performed on CD1 mice (Charles River). We used *Gad67-Gfp* [G42 line: CB6-Tg(Gad1-EGFP)G42ZjhJ]^134^ littermates to study the P11-P13 developmental pattern of expression of PV and WFA and the P12-P13 electro-physiological recordings of PV^+^ interneurons. To investigate and compare the pattern of PV, WFA, *Ppargc1a*, and PGC-1*α* expression, we crossed *Sst^Cre/+^* mice [Sst^tm2.1(cre)Zjh^]^131^ with *Ai9* mice, and *Lhx6-Cre* mice to *RCE* mice [Gt(ROSA)26Sor^tm1.1(CAG-EGFP)Fsh^]^130^ (JAX032037). To characterize the role of PGC-1*α* in the maturation of PV^+^ interneurons, mice carrying loxP-flanked *Ppargc1a* alleles [Ppargc1a^tm2.1Brsp^]^71^ (JAX009666) were crossed to *Lhx6-Cre* or *Lhx6-Cre;RCE* mice, while *Lhx6-Cre* mice were used in *Ppargc1a* over-expression experiments. *Lhx6-Cre;Ai9* mice were used to assess the pattern of expression of MEF2c, *Esrra*, and *Esrrg. Mef2c*-floxed mice [Mef2c^tm1Jjs^]^128^ (JAX025556) were crossed to *Lhx6-Cre;Ai9* mice to study the role of Mef2c in the development of PV^+^ interneurons. *Cox6a2^-/-^* mouse knockouts were processed by the Khodosevic lab to assess the impact of Cox6a2 on PV expression and perineuronal net formation during the maturation of these cells. WT C57BL6 mice (Charles River) were used for CU-T&RUN experiments.

### Method Details

#### Viral production

Serotype 8 adeno-associated viruses (AAV8) were produced in HEK293FT cells grown on 5 (Polysciences Europe, 23966-100) or 10 15-cm diameter plates (Sigma-Aldrich #408727), depending on whether we used linear or branched polyethylenimine (PEI) transfection, respectively, until cells reached 60% confluency. Cells were grown on DMEM (Gibco #21969-035) supplemented with 10% fetal bovine serum (FBS) (Gibco #10500-064), 1% penicillin/streptomycin (Gibco #15140-122), and 10 mM HEPES. AAVs were produced using PEI transfection of HEK293FT cells with a virus-specific transfer plasmid (70μg/10 plates) and a pDP8.ape helper plasmid (300 μg/10 plates; PlasmidFactory #PF478). The helper plasmid provided the AAV Rep and Cap functions and the Ad5 genes (VA RNAs, E2A, and E4). The DNA and PEI were mixed in a 1:4 ratio in uncomplemented DMEM and left at room temperature for 25 min to form the DNA-PEI complex. The transfection solution was added to each plate and incubated for 72h at 37°C in 5% CO_2_. The transfected cells were then scraped off the plates and pelleted. The cell pellet was lysed in buffer containing 50 Mm Tris-Cl, 150 mM NaCl, 2 mM MgCl_2_, and 0.5% Sodium deoxycholate and incubated with 100 U/mL Benzonase nuclease (Sigma #E1014 25KU) for 1 h to dissociate particles from membranes. The particles were cleared by centrifugation, and the clear supernatant was filtered through 0.8 μm (Merck Millipore #SLAA033SS) and 0.45μm (Merck Millipore #SLHA 033SS) filters. The viral suspension was loaded on a discontinuous iodixanol gradient using four layers of different iodixanol concentrations^135^ of 15%, 25%, 40%, and 58% in Quick-seal polyallomer tubes (Beckman Coulter #342414) and spun in a Vti-50 rotor at 50.000 rpm for 75 min at 12°C in an Optima L-100 XP Beckman Coulter ultracentrifuge to remove any remaining contaminants. After completion of the centrifugation, 5mL was withdrawn from the 40-58% interface using a G20 needle. The recovered virus fraction was purified by first passing through a 100 kDa molecular weight cutoff (MWCO) centrifugal filter (Sartorius, VIVASPIN VS2041) and then through an Amicon Ultra 2mL Centrifugal filter (Millipore #UFC210024). Storage buffer (350 mM NaCl and 5% Sorbitol in PBS) was added to the purified virus, and 5 μl aliquots were stored at -80°C.

#### Viral injections

Pups were separated from the dam and anesthetized with 1.5% isoflurane via a nose cone and placed in a stereotaxic frame containing a heating pad. Glass capillaries with a tip diameter of 30-45 μm were attached to a Nanoinjector (Drummond). Pups were injected with specific viral mixes, depending on the experiment, at Z-depth coordinates -0.2, -0.4, -0.6 to target cells of all cortical layers and delivered in two locations of S1 of the left hemisphere at an injection rate of 5nL/s (unless specified otherwise below). Pups were allowed to recover in a heating chamber at 37°C and then returned to the dam.

#### DREADD experiments

*Lhx6-Cre* or *Lhx6-Cre;Ppargc1a^F/F^* pups were injected at postnatal day (P)0 or P1 with 600nL of the following viral mix: *AAV8-hSyndio-hM3D(Gq)-mCherry* (Addgene #44361-AAV8) at a 1:40 dilution for sparse cell targeting, mixed with *AAV8-hSyn-dio-GFP* (Addgene #50457-AAV8, 1:2 dilution with sterile PBS and 0.5% Fast Green, Sigma). Another experiment using *Lhx6-Cre;Sst^Flp/+^* mice was based on the injection of 600nL of *AAV8-hSyn-dio-frt-hM3D(Gq)-mCherry* (Addgene #217398) at a 1:40 dilution for sparse cell targeting, mixed with *AAV8-hSyn-dio-GFP* (Addgene #50457-AAV8, 1:2 dilution with sterile PBS and 0.5% Fast Green, Sigma). *Lhx6-Cre* pups were also injected at P0 or P1 in a separate experiment, with 600 nL of a viral mix composed of *AAV8-hSyn-dio-hM4D (Gi)-mCherry* (Addgene #44362-AAV8) at a 1:40 dilution for sparse cell targeting and *AAV8-hSyn-dio-GFP* (Addgene #50457-AAV8, 1:2 dilution with sterile PBS and 0.5% Fast Green, Sigma). A final series of experiments was based on the injection of 600 nL of *AAV8-hSyn-dio-hM4D(Gi)-mCherry* at a 1:40 dilution for sparse cell targeting together with *AAV8-hSyn-dio-GFP* and custom-made *AAV8-hSyn-dio-Ppargc1a* viruses. The dilution factor of the DREADDs viruses (here 1:40) was calculated to reach a titer of 8.10^11^ for sparse labeling.

#### Excitatory innervation experiments

To assess the role of excitatory innervation on the maturation of PV^+^ interneurons, *ErbB4^F/F^;Ai9* mice and *ErbB4^+/+^;Ai9* mice were injected at P1 with 600 nL of custom-made *AAV8-E2-Cre*, diluted at 1:15 with sterile PBS and 0.5% Fast Green (Sigma). The Rico lab gifted the E2-Cre construct, which was subsequently cloned into an AAV8 backbone. The dilution factor of the virus (here 1:15) was calculated to reach a titer of 8.10^11^ for sparse labeling. We observed leakage of Cre recombination in excitatory cells following injection at P1, which was ignored since ErbB4 is only expressed in putative PV interneurons at postnatal ages.

#### Glutamate release experiments

To assess the role of glutamate release by excitatory cortical neurons on the maturation of PV^+^ interneurons, *AAV8-hSyn-dio-shLacZ* or a mix (1-to-1 ratio) of *AAV8-hSyn-dio-shVglut1* and *AAV8-hSyn-dio-shVglut2* colored with 0.5% Fast Green (Sigma) were injected at P5 in *Nex^Cre/+^* mice. We used previously designed shRNA sequences to target Vglut1 and Vglut2.^136,137^ A volume of 600 nL was injected at a speed of 50 nL/min. The skin of the animals was first cut with a blade, the capillary containing the viral mix was pulled down through the soft skull at the coordinates specified above, and the skin was closed after surgery using surgical glue (3M Vetbond #1469SB). The Rico lab gifted the *AAV8-hSyn-dio-shLacZ* construct, which was subsequently used to clone the other constructs. To decrease glutamate release from thalamocortical projections, we co-injected the above viruses with *AAV8-hSyn-Cre-Gfp* viruses in the thalamus of P0 CD1 mice. A volume of 150 nL was injected at each injection point at a speed of 50 nL/min. The capillary containing the viral mix was pulled down through the soft skin and skull at the coordinates (X=1.2, Y= 0.9, Z= -2.6 and -2.7) from Lambda, and was slowly pulled out three minutes after the last injection. The brains containing mCherry^+^ thalamocortical projections were selected for further analysis.

#### ION experiments

To abolish whisker-associated somatosensation, we sectioned the infraorbital nerve at the level of the whisker pad at P9 via a single scalpel incision. The sectioned skin was closed with surgical glue (3M Vetbond #1469SB).

#### Single-cell RNA-sequencing experiments

*Lhx6-Cre;Ppargc1a^+/+^* and *Lhx6-Cre;Ppargc1a^F/F^* animals were injected in both hemispheres at P1 with 900nL of *AAV8-hSyn-dio-tdTomato* colored with 0.5% Fast Green (Sigma) (300nL injected at three different locations of S1 in each hemisphere).

#### Electrophysiology on controls and conditional Ppargc1a mutants

*Lhx6-Cre;Ppargc1a^+/+^* and *Lhx6-Cre;Ppargc1a^F/F^* animals were injected at P1 with 600nL of *AAV8-hSyn-dio-Gfp* colored with 0.5% Fast Green (Sigma) (300nL injected at three different locations of S1).

#### Ppargc1a *overexpression experiments*

*Lhx6-Cre* animals were injected at P1 with 600nL of a viral mix composed of *AAV8-hSyn-dio-Gfp* and custom-made *AAV8-hSyn-dio-Ppargc1a* colored with 0.5% Fast Green (Sigma) (300nL injected at three different locations of S1).

#### Mito-EmGFP experiments

*Lhx6-Cre;Ppargc1a^+/+^* and *Lhx6-Cre;Ppargc1a^F/F^* animals were injected at P1 with 600nL of custom-made *AAV8-hSyn-dio-mito-EmGFP* (diluted 1:2 with sterile PBS and 0.5% Fast Green, Sigma). The Vanderhaeghen lab gifted the mito-EmGFP construct, which was subsequently cloned into an AAV8 backbone.

#### CNO administration

Clozapine-N-Oxide (CNO, Tocris #4936) was dissolved in 5% dimethyl sulfoxide (Sigma) and then diluted with 0.9% saline to 0.1 mg/mL. Pups were injected intraperitoneally with vehicle (0.05% DMSO) or CNO (10 μL/g) twice daily for two days and a half (from P10 to P12). Vehicle and CNO treatments were randomly assigned among littermates.

### Histology

#### Immunohistochemistry

Mice were anesthetized with an overdose of sodium pentobarbital and transcardially perfused with saline, followed by 4% parafor-maldehyde (PFA). Brains were postfixed 2h at 4°C, cryoprotected in 15% sucrose followed by 30% sucrose, and sectioned frozen on a sliding microtome at 40 μm or 60 μm. All primary and secondary antibodies were diluted in PBS containing 0.25% Triton X-100, 10% horse serum, and 2% BSA. The following antibodies were used: goat anti-mCherry (1:500, Antibodies Online #ABIN1440058), chicken anti-GFP (1:1000, Aves Labs #GFP-1020), rabbit anti-parvalbumin (1:5000, Swant #PV-27), guinea pig anti-parvalbumin (1:1,000, Synaptic Systems #195-004), Wisteria Floribunda Agglutinin (1:200, Sigma Aldrich #L-1516), rabbit anti-somatostatin (1:3,000, BMA Biomedicals #T-4103), mouse anti-PGC-1*α* (1:200, Santa Cruz #sc-518025), mouse anti-Kv1.1 (1:1,000, Antibodies Incorporated #75-105), mouse anti-Kv3.1 (1:1,000, Antibodies Incorporated #75-041), mouse anti-ERRγ (1:100, R&D Systems #PP-H6812-00), rabbit anti-MEF2c (1:100, ProteinTech #18290-1-AP), rabbit anti-Fos (1:500, Cell Signaling #2250). We used Alexa Fluor-conjugated (1:400, ThermoFisher), donkey-anti-guinea pig 647 (1:250, Jackson ImmunoResearch #706-605-148), donkey-anti-chicken 488 (1:200, Jackson ImmunoResearch #703-545-155), donkey anti-rabbit 405 (1:200, Abcam #ab175651), Streptavidin 555 (1:400, ThermoFisher #S21381) and Streptavidin 647 (1:400, Jackson ImmunoResearch #016-600-084) secondary antibodies. Sections were finally counterstained with DAPI (5 μM) and mounted with Mowiol-Dabco mounting medium (Sigma).

#### Single-molecule fluorescent in situ hybridization

All single-molecule fluorescent *in situ* hybridization solutions were prepared in RNAse-free water and PBS. Mice were perfused as described above, and brains were postfixed overnight at 4°C, cryoprotected in 15% sucrose followed by 30% sucrose, and sectioned frozen on a sliding microtome at 30 μm. Sections were mounted on RNAse-free SuperFrost Plus slides (ThermoFisher) and probed against target RNAs using the RNAscope Multiplex Fluorescent Assay v2 according to the manufacturer’s protocol (ACDbio #323110). The following probes were used: Mm-*Lhx6*-C2 (#422791-C2), Mm-*Lhx6*-C3 (#422791-C3), Mm-*Sst*-C3 (#404631-C3), Mm-*Ppargc1a*-C1 (#402121), Mm-*Bcan*-O2-C2 (#531761-C2), Mm-*Cox6a2*-C3 (#461371-C3), Mm-*Esrra*-O2-C2 (#534441-C2), Mm-*Esrrg*-C3 (#495121-C3), Mm-*Reln*-C2 (#405981-C2), Mm-*Tac1*-C3 (#410351-C3), Mm-*Slc17a6-C2* (#319171-C2), Mm-*Slc17a7-C1* (#416631). When required, the RNAscope protocol was then followed by immunohistochemistry against mCherry (goat anti-mCherry, 1:250, Antibodies Online) to stain cells infected with adeno-associated viruses expressing mCherry or against GFP (using a combination of two rabbit anti-GFP antibodies, 1:500, Aves Labs #GFP-1020 and Abcam #ab13970) to reveal the endogenous GFP signal of MGE-derived cells labeled with an RCE reporter. Mounted sections were washed with PBS and blocked for 30 min in PBS containing 10% horse serum and 2% BSA. The sections were then incubated overnight at 4°C with the primary antibody. The next day, the sections were washed three times in PBS and incubated with a secondary antibody for 2 h at room temperature. Mounted sections were finally counterstained with DAPI (5 μM) before drying and applying Mowiol-Dabco mounting medium (Sigma). Primary and secondary antibodies were diluted in PBS with 10% horse serum and 2% BSA.

### Single-cell RNA sequencing

#### Preparation of single-cell suspension

*Lhx6-Cre;Ppargc1a^+/+^* and *Lhx6-Cre;Ppargc1a^F/F^* animals, injected in both hemispheres with *AAV8-hSyn-dio-tdTomato* viruses at P1, were processed separately in three control and three mutant batches (control batch 1: 2 animals; control batch 2: 2 animals; control batch 3: 2 animals; mutant batch 1: 1 animal; mutant batch 2: 3 animals; mutant batch 3: 2 animals). For each animal, the somatosensory cortex of both hemispheres was dissociated into a single-cell suspension. Mice were deeply anesthetized with sodium pentobarbital by intraperitoneal injection and transcardially perfused with ice-cold oxygenated cutting solution (87mM NaCl, 2.5mM KCl, 1.25mM NaH_2_PO_4_, 26 mM NaHCO_3_, 75mM sucrose, 20mM D-glucose, 0.5mM CaCl_2_*2H_2_O, 4mM MgSO_4_*7H_2_O). The brain was extracted and incubated for three minutes in an ice-cold oxygenated cutting solution. The somatosensory cortex was then dissected into chunks in icy oxygenated cutting solution, and the tissue was dissociated using the Papain Dissociation System (Worthington #LK003160). After 30 minutes of incubation at 37°C with papain solution, the tissue chunks were washed with an oxygenated cutting solution and were manually and slowly dissociated using a 10mL pipette and a 1mL pipette tip. All solutions used during this process were oxygenated for at least 10 minutes with 95% O_2_ and 5% CO_2_. After papain incubation, all steps were performed on ice and using low retention filter tips (VWR). Oxygenation and a short time of dissection are crucial for the viability of the cells in suspension. After dissociation, small tissue chunks may remain, and these were discarded by filtering through a 100μm cell strainer (Fisher Scientific #11587522). The debris were removed by overlaying the single-cell suspension on top of a 10% Percoll solution (Sigma #P4937, diluted in oxygenated cutting solution and 1.5M NaCl). After a 10-min 500g centrifugation at 4°C, the pelleted single-cells were resuspended in cutting solution with 2% BSA, filtered through a 70μm cell strainer (Fisher Scientific #11597522), and transferred into a FACS tube (Falcon #352063) precoated with 3% BSA overnight.

#### Fluorescence-activated cell sorting

Samples were sorted at 4°C on BD FACS Aria II or Aria III Cell sorters for no longer than 40 min and using a 130 μm nozzle to increase cell viability. DAPI was added to the single-cell suspension to discard dead cells. Viable tdTomato^+^ single cells were collected into 1 mL of cutting solution with 2% BSA in LoBind tubes (SLS #Z666505) precoated with 3% BSA overnight. Collection tubes were centrifugated for 5 min at 4°C at 500 g, and the supernatant was discarded until 45 μL were left in the tube. Cells were resuspended, and cell number and viability were assessed using Trypan Blue and a hemacytometer for cell counts. We only carried out single-cell RNA-sequencing in experiments with viability above 60%. Between 3,000-5,000 cells were loaded for each sample onto a 10X Genomic single-cell chip/machine for single-cell capture and cDNA library preparation. RNA-seq was performed in an Illumina NextSeq 2000 platform.

### Bioinformatic analyses of single-cell and bulk RNA-sequencing

#### Data pre-processing

Illumina BCL output files were demultiplexed into FASTQs and used as inputs to the Cell Ranger count pipeline (v7.0.1). Alignment was performed to the mm10 genome, and intronic reads were excluded, but the pipeline was otherwise run with default parameters. The filtered feature barcode matrix was used as input for further analysis in Seurat (v4.3.0) in Rstudio (v2023.06.2+561).

#### Quality control

Upon initial creation of the Seurat object, genes detected in fewer than 3 cells and cells with fewer than 200 reads were discarded. Mitochondrial, ribosomal, and sex-specific genes were not excluded. In subsequent quality control, cells were retained if they had <10% ribosomal reads. Lower and upper limits for unique gene counts and lower limits for the percentage of mitochondrial reads were established for each sample individually (Figure S4). The total number of cells retained after filtering were 1489, 2797, and 982 cells for control replicates and 772, 991, and 1376 for mutant replicates.

#### Normalization, scaling, and variable feature detection

Cell cycle scores were calculated using the *CellCycleScoring* function. Sample read counts were subsequently normalized and variance-stabilized using *SCTransform*, regressing out percentages of mitochondrial and ribosomal reads and cell cycle score difference (calculated by subtracting the G2M score from the S score). This function was also used to identify the top 3000 variable features.

#### Data integration

Replicates within each condition were merged, and the pooled control and mutant datasets were individually subjected to *SCTransform* normalization. The same variables were regressed as above. The control and mutant datasets were then integrated according to the *SCTransform* integration workflow (*SelectIntegrationFeatures* with the top 3,000 variable features, *PrepSCTIntegration*, followed by *FindIntegrationAnchors* and *IntegrateData* specifying SCT as the normalization method).

#### Dimension reduction and clustering

Dimension reduction and clustering were carried out using the standard Seurat workflow (*RunPCA* with all variable features, *RunUMAP* and *FindNeighbors* with all 50 dimensions, and *FindClusters* at 1.5 resolution). Markers for each cluster were computed with the *FindAllMarkers* function. Only genes expressed in >10% of cells in at least one of the populations being compared were considered, and markers with adjusted *p* < 0.05 were taken to be differentially expressed. Of 27 clusters, 7 pertaining to non-neuronal or other undesired populations were excluded. The remaining clusters could be assigned PV^+^ or SST^+^ interneuron identity based on the expression of *Pvalb* and *Sst*, yielding a total of 1,376 and 995 control and mutant PV^+^ interneurons, and 3,098 and 2029 control and mutant SST^+^ interneurons, respectively. The control and mutant datasets were then split, *SCTransformed*, re-integrated according to the *SCTransform* integration workflow, and subjected to dimension reduction (*RunPCA* with all variable features, *RunUMAP* with 50 dimensions). We also performed label transfer with canonical correlation analysis (CCA) dimension reduction and SCT normalization using the PV^+^ and SST^+^ cells from Tasic and colleagues^64^ to verify our previous cluster assignment analysis. The two methods produced the same results, and we used the cluster-by-cluster assignment for all our subsequent analyses.

#### Differential gene expression analysis

DGE analysis was performed on RNA counts normalized according to default parameters, employing the *FindMarkers* or *FindAllMarkers* functions in Seurat. Unless specified otherwise, only genes expressed in >10% of cells in at least one of the populations being compared, and with average log_2_ fold change >|0.25| and adjusted p-value < 0.05, were considered. To find PV and SST-enriched genes at P21, we compared wild type PV^+^ and SST^+^ interneurons in our own dataset, using a log_2_ fold change threshold of >|0.35|. To find mature markers of different interneuron subclasses, we performed DGE analysis using a single-cell RNA-sequencing dataset from the adult mouse cortex^64^ and used a log_2_ fold change threshold of >|0.45|. To identify genes enriched in L4 and L5/6 PV^+^ interneurons, we used the Patch-seq dataset from the adult mouse cortex^83^ to infer the layer localization of different transcriptomic subtypes. Layer assignments were generalized to the entire Patch-seq dataset, but Chandelier cells (*Pvalb Vipr2*) and subtypes that could not be assigned to any layer (*Pvalb Calb1 Sst, Pvalb Gpr149 Islr*) were excluded from the analysis. PV^+^ interneuron subtypes assigned to L4 and L5/6 were used for head-to-head DGE analysis.

#### Assignment of layer identities

As described above, we used a Patch-seq dataset^83^ to infer layer localization of different PV^+^ interneuron subtypes. Read counts were subjected to *SCTransform* normalization as described for the P21 dataset. Layer identities were assigned from the reference dataset by label transfer (*FindTransferAnchors*, followed by *TransferData*), specifying 50 dimensions, *SCTransform* as the normalization method, and employing CCA dimension reduction under the assumption that all cortical layers are represented in our dataset.

#### Pathway enrichment analysis

Pathway enrichment analysis was performed with the *compareCluster* function from the *clusterProfiler* package (v4.4.2), using Reactome pathway terms.

#### Selection of candidate transcription factors and cofactors as regulators of PV^+^ interneuron maturation

We analyzed bulk RNA sequencing datasets of PV^+^ and SST^+^ subclasses of interneurons using the SYNAPDOMAIN dataset from Favuzzi and colleagues^59^ (GEO: GSE120161). PV-specific genes were identified using differential gene expression analysis of P10 PV^+^ and SST^+^ cells and filtering for genes with a minimum threshold of expression of 10 FPKM in PV^+^ cells to exclude genes with undetectable expression levels in vivo. We then selected genes with significant upregulation of their expression levels between P5 and P10. We refined our list of candidates further to include only transcription factors and cofactors using the AnimalTFDB database (http://guolab.wchscu.cn/AnimalTFDB4/#/).

#### Selection and analysis of metabolic genes

Data was pre-processed according to the standard Seurat workflow, as described above. To identify metabolic genes upregulated in PV^+^ cells between P10 and P28, we integrated data from two single-cell RNA sequencing experiments of the ALM performed at these stages by Allaway and colleagues^69^ (GEO: GSE165233; sample accessions GSM5014305 and GSM5014307, respectively). Within the integrated dataset, PV^+^ cells were identified through label transfer with CCA dimension reduction and SCT normalization from a dataset published by Tasic and colleagues.^64^ The reference dataset was first filtered to only retain all major subclasses of interneuron. Differential gene expression analysis was performed on P10 and P28 PV^+^ cells using normalized RNA counts, a minimum log2FC threshold of 0.25, and only considering genes expressed in at least 10% of the cells of either population being compared. The resulting genes were filtered for metabolic genes using a dataset from Iwata and colleagues.^68^

### CUT&RUN

#### Preparation of nuclei suspension

The somatosensory cortex was dissected from P12 WT C57BL6 mice in ice-cold buffer HB (0.25 M sucrose, 25 mM KCl, 5 mM MgCl2, 20 mM Tricine-KOH pH 7.8, 1 mM DTT, 0.15 mM spermine, 0.5 mM spermidine). Cortices were homogenized by a Dounce homogenizer in the presence of 0.3% IGEPAL. The homogenate was then filtered through a 40 μm strainer (Fisher Scientific #11597522), centrifuged at 4°C for 5 min at 500 g, and the pellet was washed twice with 1% BSA solution. The pelleted nuclei were resuspended in CUT&RUN Wash buffer (20mM Tris-HCl pH 7.5, 150mM NaCl, 0.5mM spermidine, 0.1% BSA), and the shape of the nuclei was verified using Tryptan Blue and a hemacytometer. All steps were performed on ice and using LoBind pipette tips.

#### CUT&RUN

Nuclei were incubated with 2 μg of mouse anti-PGC-1*α* antibody (Millipore #ST1202, two replicates) or 2 μg of mouse anti-ERRγ anti-body (R&D Systems #PP-H6812-00, one replicate) in CUT&RUN Wash buffer for 10min with rotation at 4°C. 2 μg of rabbit anti-mouse IgG secondary antibodies (Abcam #ab6709) were then added to the nuclei solution, which was left to rotate for 10min at 4°C. Nuclei were then incubated without washing step with pA-MNase (1.6 μg/μl) at 4 °C with rotation for an additional 10 min. Nuclei were then washed and resuspended with CUT&RUN wash buffer. DNA-tethered cleavage was induced by adding CaCl_2_ at a final concentration of 2 mM on ice water for 10 min. The reaction was stopped by adding Stop buffer (200 mM NaCl, 20 mM EDTA, 4 mM EGTA, 50 μg/ml RNAse A (Thermo Scientific #EN0531), 40 μg/ml glycogen (Thermo Scientific #R0561)) at a 1-to-1 ratio. The shape of the nuclei was again verified using Tryptan Blue and a hemacytometer. To release the DNA fragments of interest, nuclei were incubated at 37 °C for 20 min. Solubilized chromatin of interest was separated from nuclei by centrifugation at 16,000 g at 4°C for 5 min and then digested with SDS (0.1% final concentration) and Proteinase K (0.2 mg/ml final concentration) at 70°C for 10 min. Released DNA fragments were then extracted with phenol:chloroform:isoamylalcohol (25:24:1) and 13,000 rpm centrifugation for 15 min at room temperature. The aqueous upper layer was transferred to a fresh tube, and NaCl was added at a final concentration of 250 mM. An equal volume of isopropanol was further added, and the DNA was left to precipitate for 2 h at -80°C. Precipitated DNA was then pelleted by centrifugation (13,000rpm for 15 min at 4°C), washed with 70% ice-cold EtOH, and resuspended in distilled water.

#### Library preparation

Libraries for sequencing were prepared using the NEBNext Ultra II library prep kit (New England Biolabs) following the protocol and thermocycling temperatures therein. Briefly, DNA ends from fragments harvested from the CUT&RUN experiment were repaired, 5’ ends phosphorylated and 3’ ends A tailed prior to ligation with NEBNext Adaptor, UNG was then used to linearize the ligated adapter. SPRIselect beads were used to deplete the unligated adaptor. Adaptor-ligated libraries were then barcoded by PCR following the manufacturer’s recommended protocol. Libraries were amplified for between (11-13) cycles and purified using SPRIselect beads prior to quantitation using Qubit and HS DNA tapestation. Individually barcoded libraries were then pooled and sequenced on an Illumina NextSeq 2000 instrument.

### Bioinformatic analyses of CUT&RUN

#### CUT&RUN data processing

Raw sequencing reads were trimmed using TrimGalore (v0.6.6) to remove adapters and low-quality reads. The trimmed fastq files were aligned to the mm10 genome using bowtie (v2.2.9) with the following parameters: –end-to-end –very-sensitive –no-mixed –no-discordant –phred33 -I 10 -X 700. PCR duplicates were removed using Picard (v2.6.0), and bam and fragment bed files were generated using Samtools (v1.3.1) and Bedtools (v2.25.0), selecting fragment lengths under 1,000 bp. Peaks were called for the two replicates of mouse anti-PGC-1*α* antibody (Millipore #ST1202) and the replicate of mouse anti-ERRγ antibody (R&D Systems #PP-H6812-00), using SEACR (Sparse Enrichment Analysis for CUT&RUN; v1.3), based on fragment bedgraph files generated with Bedtools *genomecov*. SEACR peaks were called as follows: SEACR_1.3.sh input_bedgraph 0.005 non stringent out_name. PGC-1*α* peaks were retained if they were captured in both replicates. All peaks overlapping with mm10 blacklisted regions (ENCODE file ID: ENCFF547MET) were removed prior to further analysis. Transcription factor motifs were identified with HOMER (Hypergeometric Optimization of Motif EnRichment; v4.10) as follows: findMotifsGenome.pl input_bed mm10 out_dir -size given. CUT&RUN peaks on the mm10 genome were visualized using the IGV (v2.16.0) software.

#### Gene set enrichment analysis

To derive targets from CUT&RUN data, peaks were annotated to genes using the annotatePeak function from the ChIPseeker package (v1.32.0), using the TxDb.Mmusculus.UCSC.mm10.knownGene and org.Mm.eg.db databases and specifying promoter regions within 3000bp on either side of the transcription start site (TSS). Genomic targets of PGC-1*α* and ERRγ were annotated as promoters, introns, exons, UTRs, or distal intergenic.

### Electrophysiology

Mice were anesthetized with an overdose of sodium pentobarbital before decapitation. Coronal slices (300μm) were cut using a VT1200S vibratome (Leica) in ice cold artificial cerebrospinal fluid (ACSF) containing (in mM): 87 NaCl, 11 glucose, 75 sucrose, 2.5 KCl, 1.25 NaH2PO4, 0.5 CaCl2, 7 MgCl2, 26 NaHCO3, oxygenated with 95% O2 and 5% CO2 and incubated for 45 minutes at 32°C and subsequently at room temperature. Slices were transferred to the recording setup 15 minutes prior to recording and incubated at 32 °C while being continuously oxygenated with 95% O2 and 5% CO2 in recording ACSF containing (in mM): 124 NaCl, 1.25 NaH2PO4, 3 KCl, 26 NaHCO3, 10 Glucose, 2 CaCl2, 1 MgCl2. Pipettes (3–5 MΩ) were made from borosilicate glass capillaries using a PC-10 pipette puller (Narishige) and filled with intracellular solution containing (in mM): 130 potassium-gluconate, 5 KCl, 10 HEPES, 2.5 MgCl2, 4 Na2ATP, 0.4 Na3GTP, 10 sodium-phosphocreatine, 0.6 EGTA (pH 7.2–7.3, 285–295 mOsm). Traces were recorded using a Multiclamp 700B amplifier (Molecular Devices), sampled at 20kHz or 50 kHz, and filtered at 3 kHz.

CNO-induced depolarization of cells expressing excitatory DREADDs (hM3Dq^+^) was tested by CNO bath application. Cells were recorded in a cell-attached configuration using a pipette containing recording ACSF using a loose seal (~100MΩ). CNO (final concentration of 10μM) was applied to the circulation recording ACSF and took ~2.5 min to reach the bath.

Intrinsic properties were analyzed using Clampfit 10.2. Intrinsic properties were defined and analyzed: RMP, the membrane potential recorded immediately after entering whole-cell configuration. All other properties were recorded from a V_hold_ of -60mV. Input resistance was calculated from 5 increasing 10pA current steps. Ih sag, calculated from the average membrane potential at the end of a 1s current pulse, initially hyperpolarizing the cell to –100 mV. The rise time is the time between 20% and 80% of the amplitude of the rising slope of the action potential. The decay time is the time between 80% and 20% of the amplitude of the decaying slope of the action potential. Half-width is the duration between half-maximum amplitude on the rising and decaying slopes. APmax is the maximum value reached by the AP. MFF is the maximum AP frequency elicited by increasing 100pA steps. fAHP is the potential difference between the threshold and minimum AP-value. Capacitance is calculated from the membrane time constant and input resistance. Experiments were conducted on G42 mice at P12 and P13 to compare the maturation of the electrophysiological properties with PV and WFA levels in PV^+^ interneurons. To assess the role of PGC-1*α* in the functional maturation of PV^+^ interneurons, *Lhx6-Cre;Ppargc1a^+/+^* and *Lhx6-Cre;Ppargc1a^F/F^* mice in which we injected a Cre-dependent GFP reporter to label PV^+^ and SST^+^ interneurons were analyzed at P21. Another set of experiments was conducted at P21 in *Lhx6-Cre* mice in which we injected Cre-dependent AAV8 viruses expressing *Ppargc1a* and a GFP reporter labeling PV^+^ and SST^+^ interneurons. Cells were considered PV^+^ interneurons when they had a maximum firing frequency over 100 Hz.

### Biochemistry

#### Nuclear fractionation

Cortices were rapidly dissected in ice-cold PBS, homogenized in cytosol lysis buffer (10 mM Hepes, 40 mM KCl, 3 mM MgCl_2_, 5% glycerol, 0.2% NP40, 1 mM DTT, cOmplete protease inhibitors, Sigma-Aldrich) and incubated for 30 min at 4°C with rotation. Following centrifugation at 500 g for 5 min at 4°C, the supernatant containing the cytosolic proteins was denatured at 80°C for 10 min in Laemmli sample buffer (80 mM Tris-HCl pH 6.8, 100 mM DTT, 8.7% glycerol, 2% SDS, 0.01% bromophenol blue). The pellet containing the nuclear proteins was washed in cytosol lysis buffer, resuspended in nuclear lysis buffer (20 mM Hepes, 420 mM NaCl, 1.5 mM MgCl_2_, 25% glycerol, 0.2 mM EDTA, 1 mM DTT, cOmplete protease inhibitors) and incubated for 30 min on ice with regular vortexing. The supernatant containing the nuclear proteins was recovered after centrifugation at 14,000 g for 10 min at 4°C. For Western blot, the nuclear fractions were denatured at 80°C for 10 min in Laemmli sample buffer. Protein concentrations were measured using Pierce 660 nm Protein Assay (ThermoFisher).

#### Co-immunoprecipitation

Nuclear fractions from P12 C57BL/6 mice were diluted in co-immunoprecipitation buffer (20 mM Hepes, 90 mM NaCl, 1 mM MgCl_2_, cOmplete protease inhibitors) and subsequently incubated overnight at 4°C with 8 μg of either rabbit anti-Mef2c (Proteintech #18290-1-AP) or rabbit IgG isotype control (Abcam #ab27478). 25 μl of Dynabeads Protein G slurry (Invitrogen) was washed in co-immuno-precipitation buffer and added to the samples for 3 h at 4°C with gentle rotation. Beads were washed six times in wash buffer (20 mM Hepes, 150 mM NaCl, 1% NP40, 1% glycerol, 1 mM MgCl_2_, cOmplete protease inhibitors), resuspended in Laemmli sample buffer, and samples were denatured at 80°C for 10 min.

#### Western blotting

20 μg of denatured protein extracts (or the whole denatured co-immunoprecipitation samples) were separated by SDS-PAGE using 10% acrylamide gels for 2 h at 120V and transferred onto methanol-activated PVDF membranes at 300 mA for 2 h on ice. Membranes were blocked with 5% non-fat milk in TBS-T (20 mM Tris-HCl pH 7.5, 150 mM NaCl, and 0.1% Tween20) for 1h at room temperature and then probed with primary antibodies in blocking buffer overnight at 4°C. The next day, membranes were washed in TBS-T and incubated with HRP-conjugated secondary antibodies for 1 h at room temperature. Depending on the amount of protein of interest in the samples, membranes were incubated with either Immobilon Western Chemiluminescent HRP substrate (Millipore), SuperSignal West Pico, or West Femto Chemiluminescent Substrates (ThermoFisher). Protein levels were visualized by chemiluminescence with an Odyssey FC (Li-Cor) and quantified with Image Studio Lite. For quantification, the densitometry of the band of interest was normalized to that of actin. The following primary antibodies were used: mouse anti-PGC-1*α* (1:200, Santa Cruz #sc-518025), rabbit anti-MEF2c (1:500, Proteintech #18290-1-AP), mouse anti-actin-peroxidase (1:20000, Sigma-Aldrich #A3854). The following HRP-conjugated secondary antibodies were used: anti-mouse-peroxidase (1:5000, Invitrogen #31444), anti-mouse-peroxidase (1:1000, Abcam #ab131368) and anti-rabbit-peroxidase (1:1000, Abcam #ab131366).

#### Luciferase assay experiments

We constructed luciferase reporter vectors by cloning promoter regions of the *Bcan, Cplx1*, and *Kcan1* genes in a pGL4.11 plasmid (Promega) by Gibson assembly (New England Biolabs) using the following primers: *Cplx1* Forward: CCAGAACATTTCTCTGGCC TCTTACTCCCCACCCCTAGC; *Cplx1* Reverse: ACAGTACCGGATTGCCAAGCGCCGGTTACCTTCC. *Bcan promoter 1* Forward: GGCCTAACTGGCCGGTACCCAGAGCGCTGCTTAGCAGG; *Bcan promoter 1* Reverse: CCAACAGTACCGGATTGCCATAGAGAC GGGTTCCTGTGTATGAAAGAC. *Bcan promoter 2* Forward: GGCCTAACTGGCCGGTACGTGTTGGCACCATCTTACATGTGG; *Bcan promoter 2* Reverse: CCAACAGTACCGGATTGCCACTTGCGAGGTCCCCTCC; *Kcna1* Forward: GGCCTAACTGGCCGG TACAGCCACTTGGGAGGATCC; *Kcna1* Reverse: CCAACAGTACCGGATTGCCACCTCAGTCCTGCCTTCCGA. HEK293T (embryonic kidney) cell lines, obtained from the American Type Culture cells, were maintained with DMEM medium supplemented with L-glutamine, 10% fetal bovine serum (Sigma-Aldrich) and 1% penicillin-streptomycin (Invitrogen). Cells were allowed to grow at 37 °C with 5% CO_2_. HEK293T cells were seeded in 12 wells plates and transfected (Fugene HD, Promega) the next day with the luciferase reporter vectors (100 ng), the expression vectors (100 ng each), and the pRL-TK plasmid to express *Renilla* as an internal control (2 ng). After 16 hours, cells were washed with PBS, and luciferase assays were performed in triplicates using the Rapid Detection Firefly Luciferase Activity assay system (Promega), following the manufacturer’s instructions, with a GloMax plate reader (Promega). Firefly luciferase activities were normalized based on the *Renilla* luciferase activity in each well.

## Quantification and Statistical Analysis

### Histological image acquisition and analysis

Images were acquired with an SP8 confocal microscope (Leica TCS SP8). Samples from the same experiment were imaged and analyzed in parallel, using the same laser power, photomultiplier gain, and detection filter settings.

#### Cell densities and proportions

Imaging was performed at 12-bit depth using a 10X objective, except for PGC-1*α* and ERRγ immunostainings where images were acquired with a 20X objective. Images following immunohistochemistry experiments were acquired with a 1024×1024-pixel resolution, while imaging of *in situ* hybridization experiments was performed with a 2048×2048-pixel resolution. Parameters of 0.75 digital zoom and 400 Hz acquisition speed were used. Between 6 and 10 sections per brain were imaged and analyzed after *in situ* hybridization or immunohistochemistry. Manual cell counting was then performed across layers of the somatosensory cortex using the FIJI (Image J) software.

#### Intensities of PV and WFA and PGC-1*α*

Imaging was performed at 16-bit depth using a 63X objective with water immersion. Images were acquired with a 2048×2048-pixel resolution, 1 or 1.5 digital zoom, and 600 Hz acquisition speed. Z-stacks were taken with a Z-step size of 1 (except for measuring PGC-1*α* levels in P10 and P12 MGE-derived cells, where a Z-step size of 2 was used) to consider the volume of a selected cell to image and analyze. A sum Z-projection was then performed for each image on FIJI.

In the excitatory DREADDs (hM3Dq-mCherry) experiments, putative PV^+^ cells in L2/3 of the somatosensory cortex labeled with mCherry or GFP were imaged, maintaining a consistent depth to the pial surface. In the absence of DAPI, L2/3 was recognized from L4 by the absence of dense PV^+^ processes. In the inhibitory DREADDs (hM4Di-mCherry) experiments, putative PV^+^ cells of L5 of the somatosensory cortex labeled with mCherry or GFP were imaged. Similarly, in the absence of DAPI, L5 was recognized from L4 and L6 by the global pattern of PV expression. A minimum of 15 DREADDs-infected mCherry^+^ cells and 15 DREADDs-un-infected GFP^+^ cells were analyzed per brain in Matlab, using a custom script. Briefly, for each image, background subtraction was applied. The contour of an mCherry^+^ prospective PV^+^ cell (identified by the lack of SST expression) was then used to draw a ROI. The mean pixel value of this ROI was measured to provide the PV intensity level of the DREADD-infected cells. PV intensity levels of DREADDs-uninfected mCherry^-^ GFP^+^ prospective PV^+^ cells (identified by the lack of SST expression) were also measured by drawing a ROI following the contour of the GFP signal. The PV intensity levels of the same numbers of DREADDs-infected and -uninfected prospective PV^+^ cells were measured per image. We calculated the median value of PV intensity levels for DREADDs-infected and -uninfected prospective PV^+^ cells separately per brain. To account for the different speeds of PV^+^ interneuron maturation between animals, PV intensity levels of DREADDs-infected prospective PV^+^ cells were normalized for each animal with the PV intensity levels of DREADDs-uninfected prospective PV^+^ cells in the same brain. These images were also used to count the number of SST^+^ cells infected by DREADDs expressing viruses (SST^+^ mCherry^+^ cells) and calculate the rate of infection of this virus in MGE-derived cells. WFA intensity levels were measured following the same methodology.

In experiments assessing the role of excitatory innervation on the maturation of PV^+^ interneurons using *ErbB4^F/F^;Ai9* mice and *ErbB4^+/+^;Ai9* mice injected with *E2-Cre* AAV8 viruses, recombined tdT^+^ PV^+^ cells in L5/6 of the somatosensory cortex were imaged. Because the expression of Cre leaks to excitatory neurons and perhaps other interneurons, we only considered PV^+^ cells that were expressing PV. We analyzed a minimum of 15 recombined tdT^+^ PV^+^ cells per brain, as well as a minimum of 15 unrecombined PV^+^ cells present in each image, using a custom Matlab script. Briefly, for each image, background subtraction was applied. The contour of PV^+^ cells in the image was drawn based on the PV signal, delineating a ROI. We identified recombined tdT^+^ PV^+^ cells and unrecombined tdT^-^ PV^+^ cells for each image. The mean pixel value of the ROI was measured to provide the PV intensity level of each PV^+^ cell (tdT^+^ and tdT^-^). We calculated the median value of PV intensity levels for tdT^+^ and tdT^-^ PV^+^ cells separately, per image, and then per brain. This value for tdT^+^ PV^+^ cells was normalized by the value for tdT^-^ PV^+^ cells per brain to account for the differences in PV interneuron maturation speed between brains. PGC-1*α* intensity levels were measured using the same methodology; only intensity levels in the nucleus (delineated using the DAPI signal) and the cytoplasm of each cell were measured.

In the ION sectioning experiments, PV intensity levels of L4 PV^+^ interneurons were compared between contralateral and ipsilateral hemispheres. The ipsilateral hemisphere is less affected by the unilateral incision of the ION and can be used as an internal control. We analyzed a minimum of 20 L4 PV^+^ interneurons per hemisphere, using a custom Matlab script. For each image, background subtraction was applied. The contour of PV^+^ cells in the image was drawn based on the PV signal, delineating a ROI. The mean pixel value of the ROI was then measured to provide the PV intensity level of each PV^+^ cell. We calculated the median value of PV intensity levels for L4 PV^+^ interneurons of each hemisphere separately, per brain. We then normalized the contralateral value to the ipsilateral value for each brain, in the ION and control (no incision) conditions.

In the experiments measuring PGC-1*α* intensity levels in prospective PV^+^ interneurons at P10 and P12 in *Lhx6-Cre;RCE* mice, these interneurons were recognized by the expression of the GFP reporter and the lack of SST expression. Putative PV^+^ interneurons of L2/3, L4, and L5 of the somatosensory cortex (20 cells per layer) were imaged and analyzed using a custom-made Matlab script. Briefly, for each image, background subtraction was applied. The somatic contour of a prospective PV^+^ cell was drawn using the GFP signal to delineate an ROI. The mean pixel value of this ROI was measured to provide the PGC-1*α* intensity level of this cell. Intensity levels in the nucleus (delineated using the DAPI signal) and the cytoplasm of each cell were also measured.

In the experiments assessing the role of Cox6a2 in the expression of PV and the formation of perineuronal nets at P12, L4 PV^+^ cells of the somatosensory cortex were identified thanks to their PV signal. We analyzed a minimum of 20 PV^+^ cells per brain, using a custom Matlab script. Briefly, for each image, background subtraction was applied. The contour of PV^+^ cells in the image was drawn based on the PV signal, delineating a ROI. The mean pixel value of the ROI was measured to provide the PV intensity level of each PV^+^ cell. To measure WFA intensity levels, a short radius outside of the ROI (and following the contour of the ROI) was analyzed. We also measured the PV and WFA intensity values of the background for each image to calculate the signal-to-noise ratio (SNR) of each measured cell. We then calculated the median value of PV and WFA SNRs separately and per brain for control and *Cox6a2* mutant mice. The SNR approach was used here as the control and experimental mutant cells belong to different animals, which can introduce variability in PV and WFA intensities due to perfusion and slicing. We only analyzed control and *Cox6a2^-/-^* mutant littermates. For all the other experiments of intensity measures described above, the SNR method was not used as the control cells are neighboring the experimental cells in the same image.

#### RNAscope dot count

For *Esrra*, and *Esrrg* mRNA particle analysis, imaging was performed at 16-bit depth using a 63X objective with water immersion. Images were acquired with a 2048×2048 pixel resolution, 1 digital zoom, and 600 Hz acquisition speed. Five S1 L2/3 and five S1 L4 ROIs were imaged per brain. *Esrra* and *Esrrg* levels were measured in a minimum of 30 MGE-derived cells per brain, which were identified by the expression of tdT. The number of *Esrra* and *Esrrg* mRNA particles was determined for all these experiments using a custom Matlab script. Briefly, background subtraction was applied to each image. The contour of tdT^+^ cells was then used to draw an ROI. The area of total *Esrra* and *Esrrg* signals was measured within each ROI. This area was divided by the area of a single mRNA particle (~0.16 μm^2^), which estimates the number of *Esrra* and *Esrrg* particles present in the ROI of interest.

#### Mito-EmGFP counts

Imaging was performed at 16-bit depth using a 63X objective with water immersion. Single-plane images were acquired with a 2048×2048 pixel resolution, 1.25 digital zoom, and 600 Hz acquisition speed. Twelve ROI in L2/3 and nine ROI in L4 of the somatosensory cortex were imaged per brain. Mito-EmGFP counts were measured in a minimum of 60 MGE-derived cells per brain (30 SST^+^ and 30 prospective PV^+^ interneurons, the latter identified by the lack of SST expression, using a custom Matlab script. Briefly, general background subtraction was applied to each image. The contour of infected cells was drawn using the mito-EmGFP signal to delineate the ROI around the soma of each cell (the script increases the brightness of the cells, boosting the GFP signal present in the cells for contour drawing only). Similarly, we drew the contours of the nucleus of each cell using the mito-EmGFP signal (this approach has been verified using the DAPI signal). We measured the levels of GFP intensity in the nucleus of each cell, which accounts for background GFP intensity levels, whose value depends on the efficacy of infection of the mito-EmGFP expressing virus per cell (more infection, more mito-EmGFP expression, more residual GFP present in the nucleus and cytoplasm). We then subtracted this background intensity value from the somatic ROI per cell. We also removed the nucleus from the somatic ROI to consider only a cytoplasmic ROI. Within each cytoplasmic ROI, the area occupied by the mito-EmGFP signal was measured, and the proportion of the cell cytoplasmic area occupied by the mito-EmGFP signal was calculated.

#### Synapses

Imaging was performed at 8-bit depth using a 100X objective with oil immersion. Single-plane images were acquired with a 2048×2048 pixel resolution, 2.2 digital zoom, and 600 Hz acquisition speed. We compared the density of Homer^+^ Bassoon^+^ excitatory synapses contacting the soma of tdT^+^ infected and neighboring tdT^-^ uninfected PV^+^ cells in L5/6 of *ErbB4^F/F^;Ai9* mice. Only PV^+^ cells with detectable levels of PV expression were recognized and used for quantification. Between 15 and 30 L5/6 PV^+^ cells were analyzed per brain. We used a custom macro in FIJI to measure Homer-Bassoon synaptic density. Background subtraction, Gaussian blurring, smoothing, and contrast enhancement were first applied in all channels. Cell somas were drawn manually based on intensity levels of PV staining to create a mask of the soma surface and measure its perimeter. Presynaptic and postsynaptic boutons were detected automatically based on thresholds of intensity. The threshold for Homer and Bassoon was selected from a set of random images before quantification, and the same thresholds were applied to all images from the same experiment. The “Analyze Particles” and “Watershed” tools were applied to the Homer and Bassoon channels, and a mask was generated with a minimum particle size of 0.06 to detect colocalized Homer and Bassoon puncta. These puncta were defined as synaptic boutons when located up to 0.3 μm^2^ inside or 0.4 μm^2^ outside the soma perimeter.

### Statistical analyses

All statistical analyses were performed using Prism 9 (GraphPad Software). Sample sizes were chosen based on previous publications in the field. Biological replicates (n values are different samples derived from different brains from different litters) were analyzed to assess the biological variability and reproducibility of data. Experimental mice from all genotypes or conditions were processed together. Samples were tested for normality using the Shapiro–Wilk normality test. Unless otherwise stated, data were analyzed by t-test or ANOVA followed by post hoc Tukey’s or Sidak’s analysis to compare multiple samples. Differences were considered significant when p values < 0.05. Data are presented as mean ± s.e.m. Statistical details of experiments are described in the figure legends. Table S5 summarizes all data and statistical tests except for the Luciferase experiments, which are included in Table S4.

## Supplementary Material

Supplemental information can be found online at https://doi.org/10.1016/j.cell.2025.06.029.

Supplemental information

Supplemental Table S2

Supplemental Table S3

Supplemental Table S4

Supplemental Table S5

Supplemental Figures

## Data Availability

The scRNA-seq, CUT&RUN, and analysis pipeline datasets have been deposited at the National Center for Biotechnology Information BioProjects Gene Expression Omnibus (GEO). They are accessible through GEO Series accession numbers GEO: GSE256441, GSE256442, and GSE256443. Other data are available on King’s College London Open Research Data System: https://doi.org/10.18742/29198687. Any additional information required to reanalyze the data reported in this paper is available from the lead contact upon request.

## In brief

Neuronal activity triggers PGC-1*α*-mediated transcriptional programs that drive the late maturation of PV^+^ interneurons, essential for cortical circuit function. PGC-1*α* partners with ERRγ and Mef2c to regulate gene expression in PV^+^ interneurons.

## Highlights

- The maturation of cortical PV^+^ interneurons depends on activity
- The activity-dependent expression of PGC-1*α* mediates this process
- PGC-1*α* cooperates with ERRγ and Mef2c to control PV^+^ interneuron maturation
- PGC-1*α* also regulates the diversification of PV^+^ interneuron subtypes
