# Supplementary material for: A postnatal molecular switch drives activity-dependent maturation of parvalbumin interneurons
Source: Cell. Author manuscript; Available in PMC 2026 Oct 9. (PMC7619535; doi:10.1016/j.cell.2025.06.029)
Supplement: Supplementary file 1 [file EMS219678-supplement-Supplemental_Figures.pdf]

CellPress
OPEN ACCESS

Supplemental figures

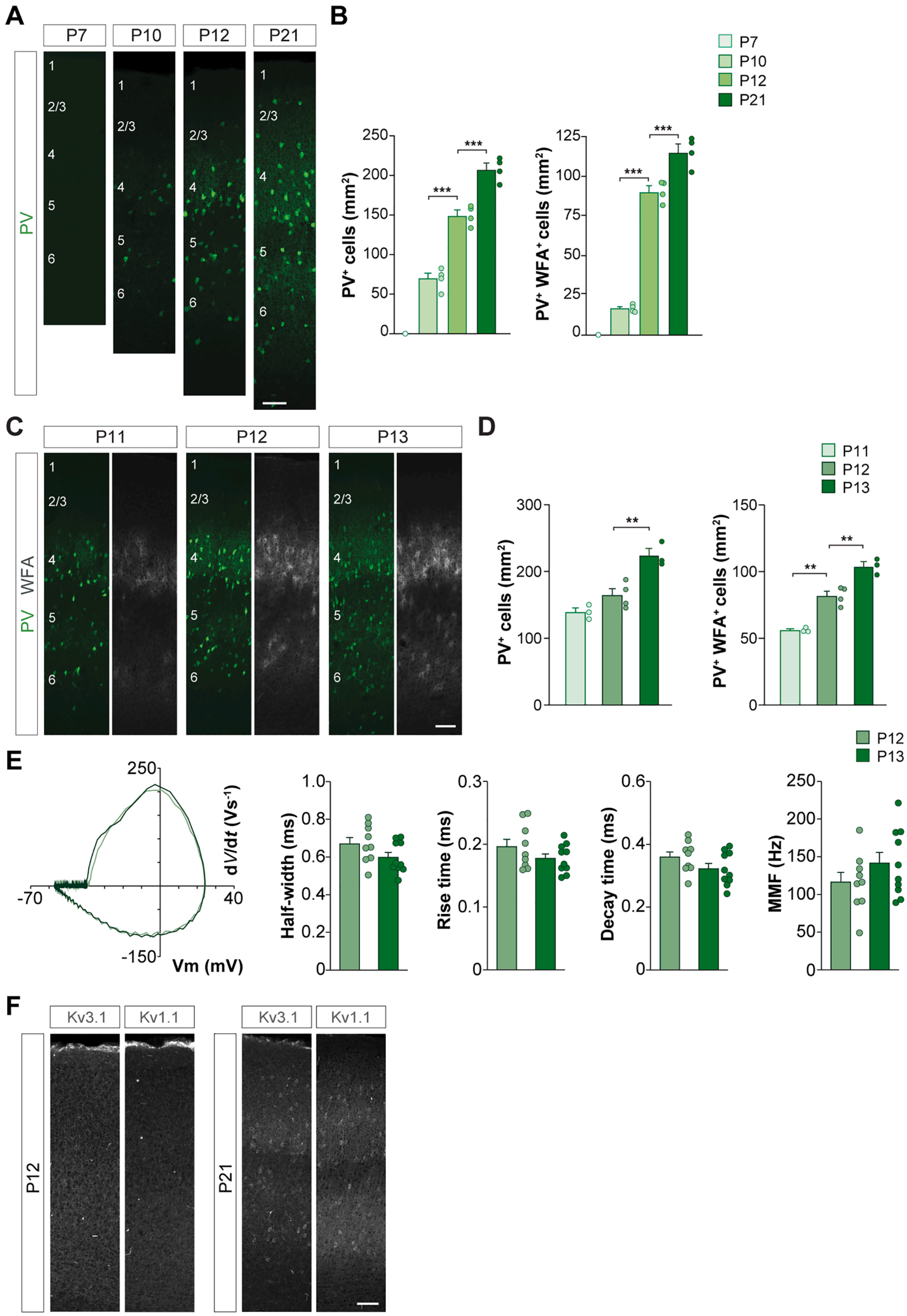

(legend on next page)

Figure S1. Maturation of S1 PV$^+$ interneurons during early postnatal development, related to Figures 1 and 2

(A) PV expression in coronal sections through S1 at P7, P10, P12, and P21.

(B) Density of PV$^+$ and PV$^+$ WFA$^+$ cells across all layers in S1 during postnatal development.

(C) PV and WFA expression in coronal sections through S1 of *G42* littermates at P11, P12, and P13.

(D) Density of PV$^+$ cells and PV$^+$ WFA$^+$ cells across all layers in S1 of *G42* littermates at P11, P12, and P13.

(E) Intrinsic properties of L5 PV$^+$ interneurons in S1 of P12 and P13 *G42* mice. Data points represent cells. MFF, maximum firing frequency.

(F) Kv3.1 and Kv1.1 expression in coronal sections through S1 at P12 and P21.

Data are presented as mean ± SEM. Scale bars, 100 μm (A, C, and F). $^{**}p < 0.01$, $^{***}p < 0.001$. See Table S5 for details on statistics.

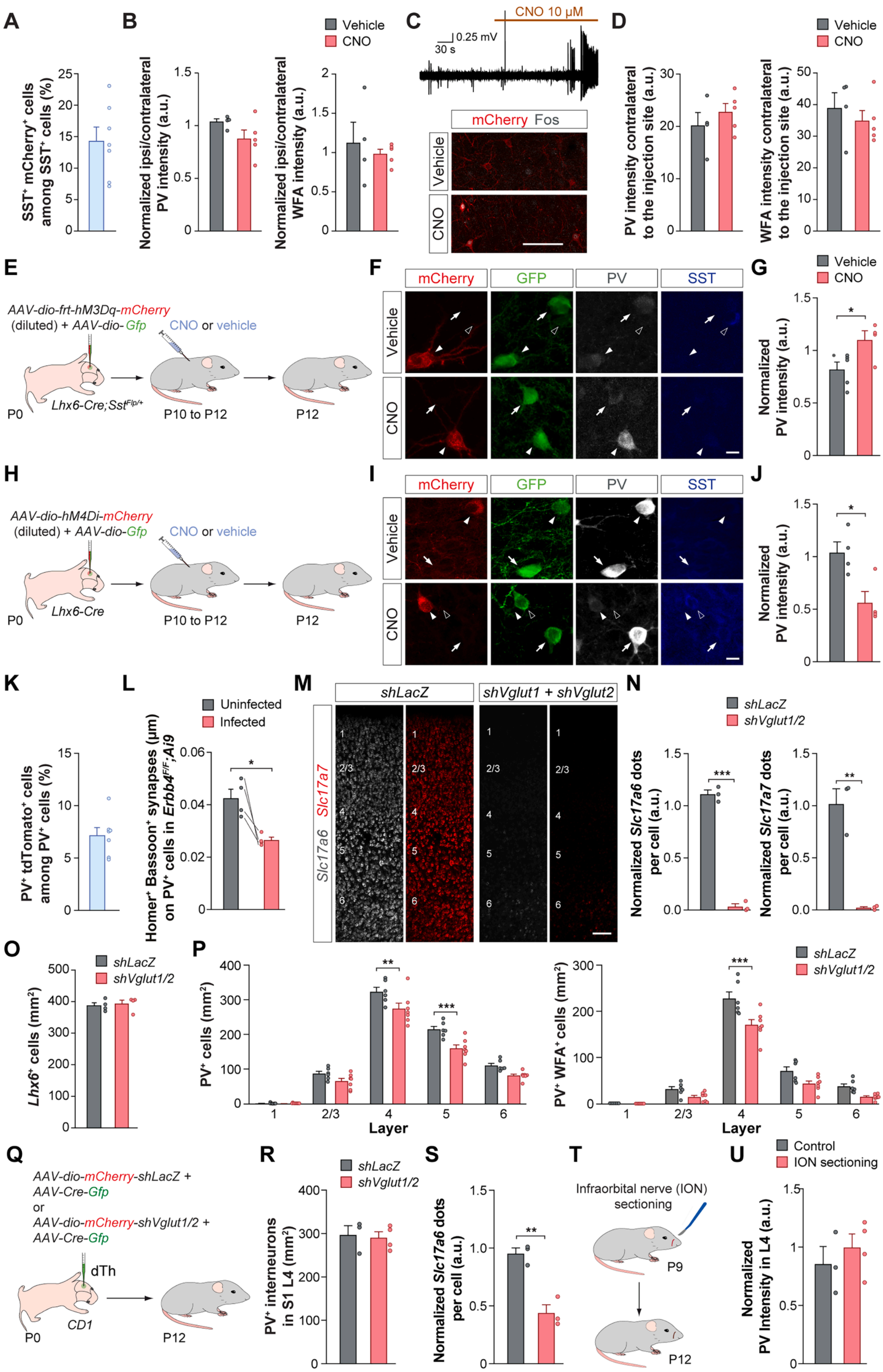

(legend on next page)

Figure S2. PV and WFA expression in prospective PV+ interneurons depends on cortical neuronal activity, related to Figure 1

(A) Infection rate of S1 L2/3 SST+ cells by *AAV8-hSyn-dio-hM3Dq-mCherry*.

(B) Detectable PV and WFA levels in S1 L2/3 PV+ interneurons on the ipsilateral side of injection, normalized to detectable PV and WFA levels on the contralateral side of injection, in control (vehicle) and experimental (CNO) conditions at P12.

(C) Electrophysiological trace of an hM3Dq+ cell before and after CNO application in the experimental bath. The images illustrate Fos expression in S1 hM3Dq+ cells of experimental (CNO) mice compared with control (vehicle) mice.

(D) Detectable PV and WFA levels in S1 L2/3 PV+ interneurons on the contralateral side of injection in control (vehicle) and experimental (CNO) conditions at P12.

(E) Experimental design.

(F) PV expression in S1 L2/3 prospective PV+ cells (recognized by the absence of SST expression) expressing mCherry or GFP in control (vehicle) and experimental (CNO) conditions at P12. Arrows point at GFP+ PV+ cells, arrowheads at mCherry+ PV+ cells, and open arrowheads at GFP+ SST+ cells.

(G) PV levels in S1 L2/3 mCherry+ prospective PV+ cells normalized by PV levels in neighboring mCherry− GFP+ prospective PV+ cells at P12.

(H) Experimental design.

(I) PV expression in S1 L5 prospective PV+ cells (recognized by the absence of SST expression) expressing mCherry or GFP in control (vehicle) and experimental (CNO) conditions at P12. Arrows point at GFP+ PV+ cells, arrowheads at mCherry+ PV+ cells, and open arrowheads at GFP+ SST+ cells.

(J) PV levels in S1 L5 mCherry+ prospective PV+ cells normalized by PV levels in neighboring mCherry− GFP+ prospective PV+ cells at P12.

(K) Infection rate of S1 L5/6 PV+ cells by *AAV8-E2-Cre*.

(L) Density of Homer+ Bassoon+ synapses contacting the soma of L5 tdTomato+ PV+ cells compared with neighboring uninfected PV+ cells in *ErbB4$^{F/F}$;Ai9* mice at P12.

(M) *Slc17a6* and *Slc17a7* expression in coronal sections through S1 from control (*shLacZ*) and experimental (*shVglut1 + shVglut2*) mice at P12.

(N) Number of *Slc17a6* or *Slc17a7* dots per cell in L5 of the infected S1 normalized to the number of *Slc17a6* or *Slc17a7* dots per cell in L5 of the neighboring uninfected somatosensory cortex from control (*shLacZ*) and experimental (*shVglut1 + shVglut2*) mice at P12.

(O) Density of *Lhx6*+ cells across all layers in S1 from control (*shLacZ*) and experimental (*shVglut1 + shVglut2*) mice at P12.

(P) Density of PV+ and PV+ WFA+ cells in each layer of S1 from control (*shLacZ*) and experimental (*shVglut1 + shVglut2*) mice at P12.

(Q) Experimental paradigm.

(R) Density of L4 PV+ interneurons in the barrel regions of S1 receiving mCherry+ thalamocortical projections at P12 following downregulation of Vglut1 and Vglut2 in the dorsal thalamus, compared with control (*shLacZ*).

(S) Number of *Slc17a6* dots per cell in the infected ventral posteromedial nucleus normalized to the number of *Slc17a6* dots per cell in the neighboring uninfected ventral posteromedial nucleus from control (*shLacZ*) and experimental (*shVglut1 + shVglut2*) mice at P12.

(T) Experimental paradigm.

(U) PV intensity in cortical L4 PV+ interneurons at P12 following ION sectioning at P9. PV intensity was measured in the contralateral side of the sectioned ION, and the ipsilateral side of the sectioned ION was used as an internal control for comparison in each animal and was used to normalize PV intensities per animal.

Data are presented as mean ± SEM. Scale bars, 100 μm (C and M) and 10 μm (F and I). *$p < 0.05$, **$p < 0.01$, ***$p < 0.001$. See Table S5 for details on statistics.

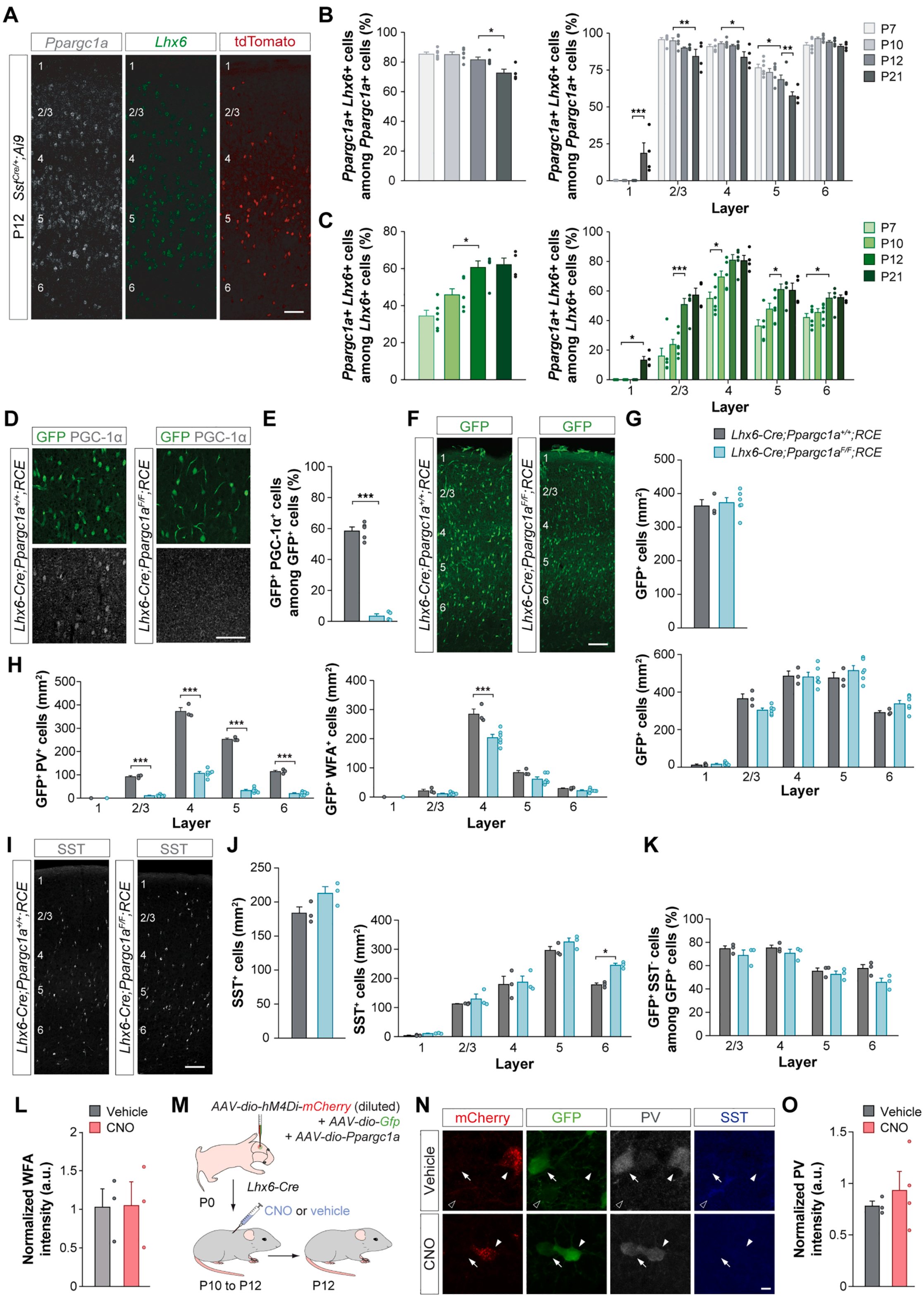

(legend on next page)

Figure S3. The onset of *Ppargc1a* expression precedes the onset of PV and WFA expression in cortical PV^+ interneurons, which depends on the activity-dependent expression of PGC-1α, related to Figure 2

(A) *Ppargc1a*, *Lhx6*, and tdTomato expression in coronal sections through S1 of $Sst^{Cre/+}$*;Ai9* mice at P12.

(B) Proportion of *Ppargc1a*$^+$ cells being *Lhx6*$^+$ in all cortical layers at P7, P10, P12, and P21.

(C) Proportion of *Lhx6*$^+$ cells being *Ppargc1a*$^+$ in all cortical layers at P7, P10, P12, and P21.

(D) PGC-1α expression in L2-4 GFP^+ cells in S1 of control and conditional *Ppargc1a* mutant mice at P21.

(E) Proportion of GFP^+ PGC-1α$^+$ cells in L2-4 of S1 in control and conditional *Ppargc1a* mutant mice at P21.

(F) GFP expression in coronal sections through S1 of control and conditional *Ppargc1a* mutant mice at P12.

(G) Density of GFP^+ cells across all layers in S1 of control and conditional *Ppargc1a* mutant mice at P12.

(H) Density of GFP^+ PV^+ cells and GFP^+ WFA^+ cells in cortical layers of S1 in control and conditional *Ppargc1a* mutant mice at P12.

(I) SST expression in coronal sections through S1 of control and conditional homozygous *Ppargc1a* mice at P12.

(J) Density of GFP^+ SST^+ cells in cortical layers of S1 in control and conditional *Ppargc1a* mutant mice at P12.

(K) Proportion of GFP^+ SST^- (i.e., prospective PV^+ interneurons) in cortical layers of S1 in control and conditional *Ppargc1a* mutant mice at P12.

(L) WFA levels in S1 L2/3 $mCherry^+$ prospective PV^+ cells normalized by WFA levels in neighboring $mCherry^-$ GFP^+ prospective PV^+ cells at P12.

(M) Experimental design.

(N) PV expression in S1 L5 prospective PV^+ cells (recognized by the absence of SST expression) expressing mCherry or GFP in control (vehicle) and experimental (CNO) conditions at P12, combined with *Ppargc1a* overexpression in both conditions. Arrows point at GFP^+ PV^+ cells, arrowheads at $mCherry^+$ PV^+ cells, and open arrowheads at GFP^+ SST^+ cells.

(O) PV levels in S1 L5 $mCherry^+$ prospective PV^+ cells normalized by PV levels in neighboring $mCherry^-$ GFP^+ prospective PV^+ cells in control (vehicle) and experimental (CNO) conditions at P12 when *Ppargc1a* is overexpressed.

Data are presented as mean ± SEM. Scale bars, 10 μm (D and N) and 100 μm (A, F, and I). *$p < 0.05$, **$p < 0.01$, ***$p < 0.001$. See Table S5 for details on statistics.

CellPress
OPEN ACCESS

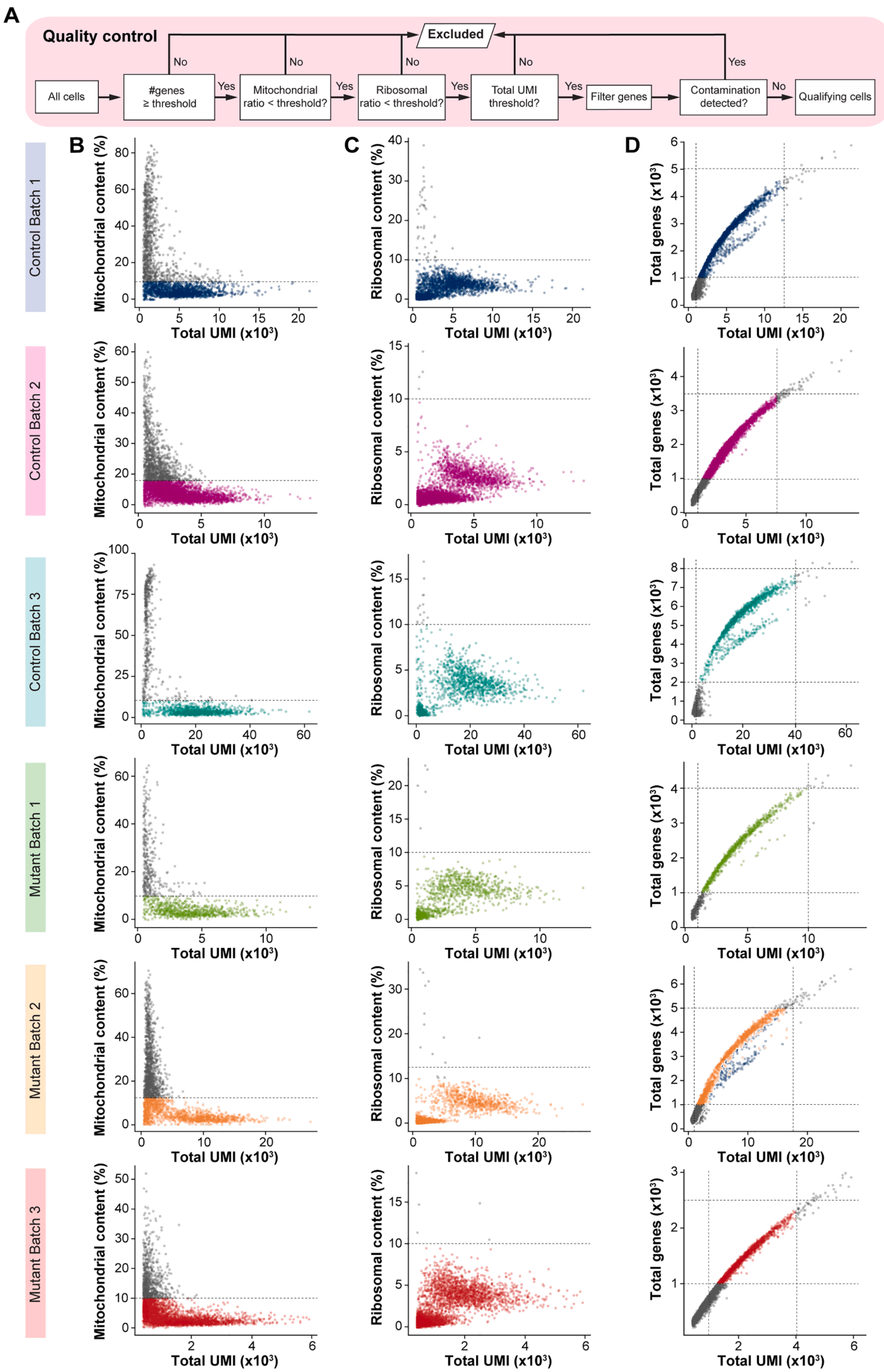

(legend on next page)

Figure S4. Quality control in single-cell RNA sequencing experiments, related to Figure 3

(A) Quality control pipeline.

(B and C) Percentage of mitochondrial and ribosomal content in each cell in each control and mutant batch of cells (UMI, unique molecular identifier).

(D) Total number of genes detected in each cell in each control and mutant batch.

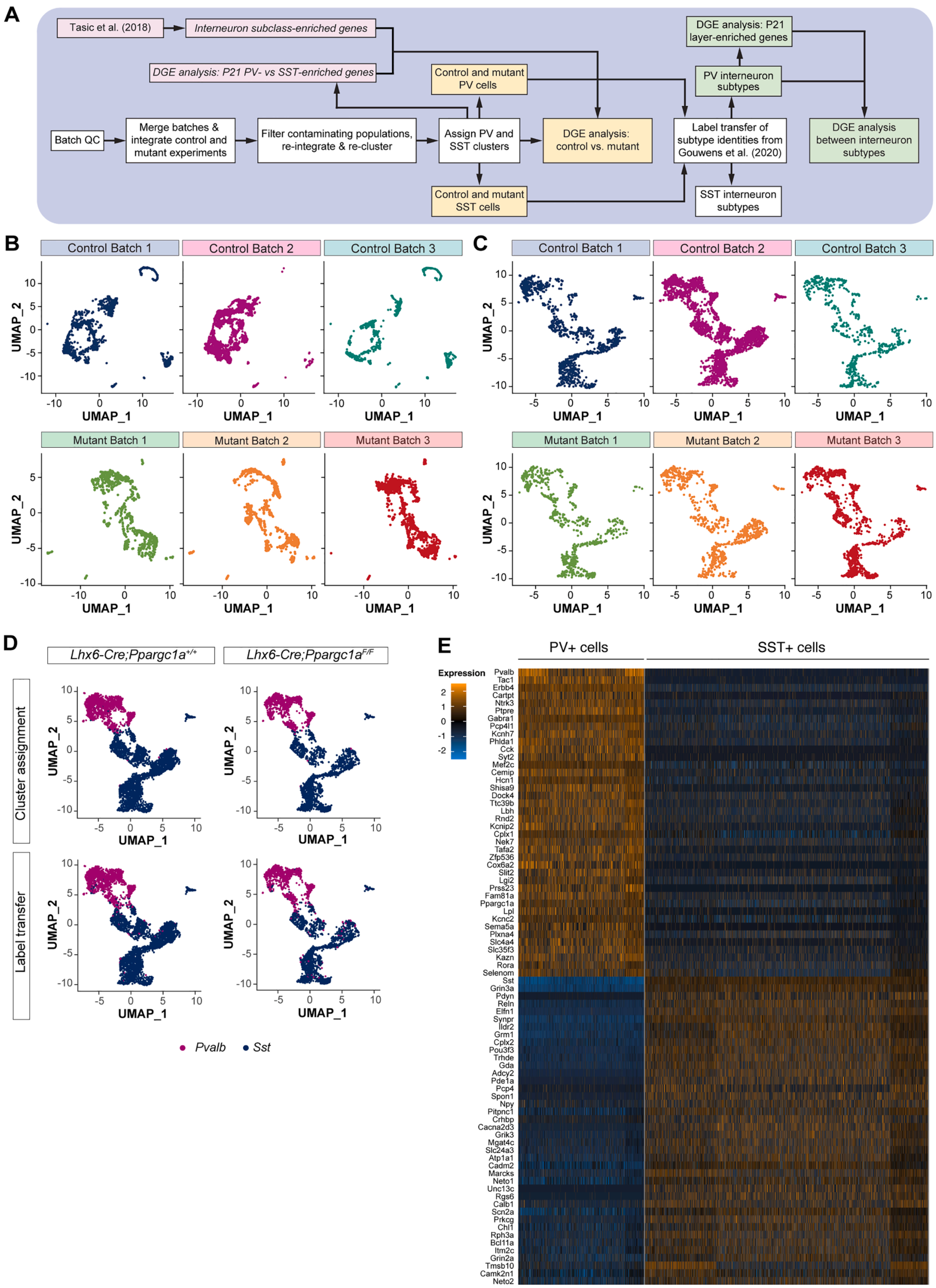

(legend on next page)

Figure S5. Gene expression alterations in PV⁺ and SST⁺ interneurons lacking PGC-1α, related to Figure 3

(A) Analysis pipeline following single-cell RNA sequencing of control and conditional homozygous *Ppargc1a* mice at P21.

(B) UMAP plots depicting the cells sequenced in the different control and mutant batches before integration of all batches.

(C) UMAP plots depicting the cells sequenced in the different control and mutant batches after integration of all batches.

(D) UMAP plots illustrating the assignment of PV^+ and SST^+ cell identities through cluster assignment and label transfer with canonical correlation analysis (CCA) dimension reduction in control and conditional *Ppargc1a* mutant mice at P21.

(E) Heatmap representing DGE between control PV^+ and SST^+ interneurons at P21. Only the top 40 genes are represented.

CellPress
OPEN ACCESS

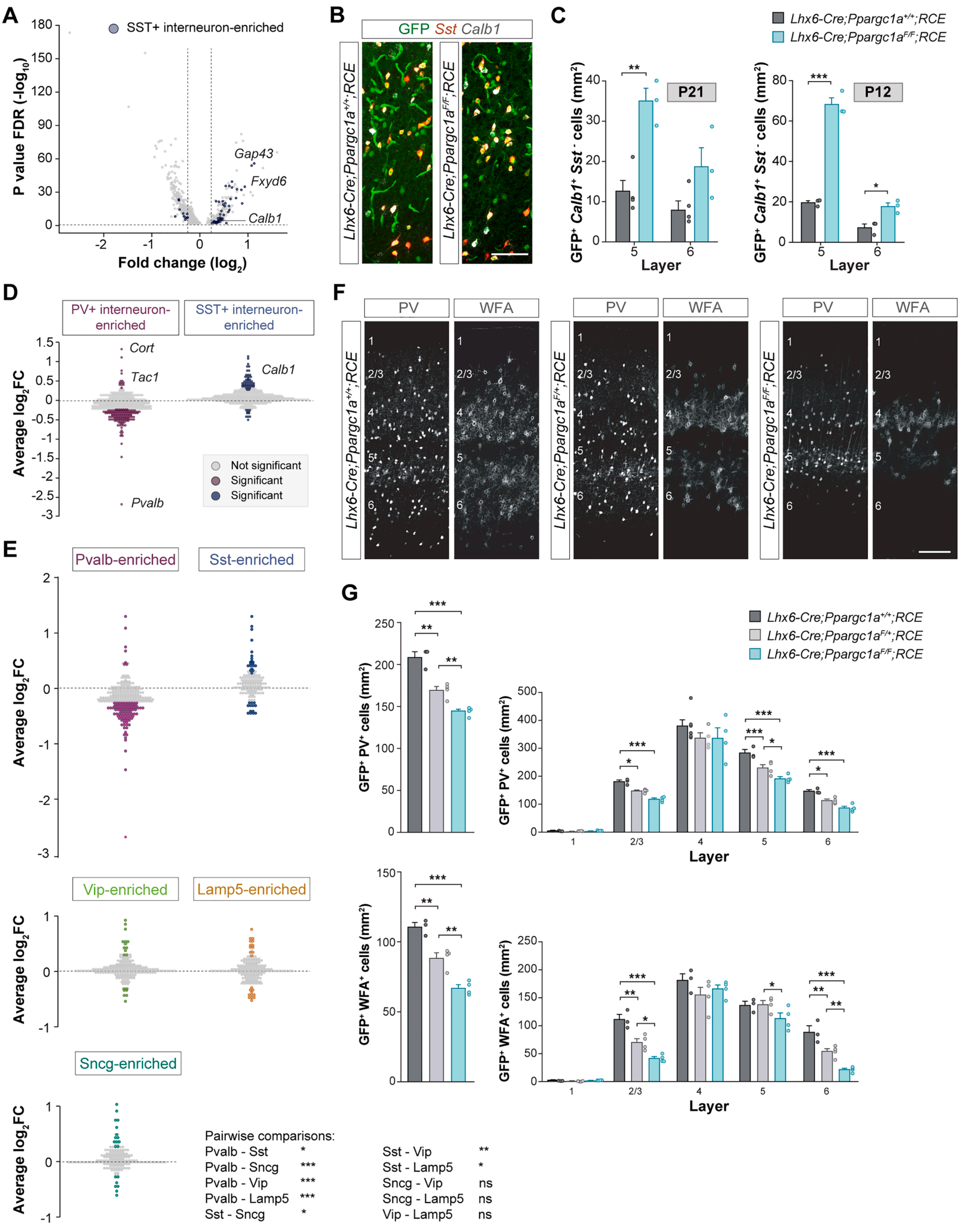

(legend on next page)

Figure S6. Loss of PGC-1α halts the expression of PV and WFA in many cortical PV+ interneurons and upregulates the expression of SST-enriched genes, related to Figure 3

(A) Volcano plot illustrating DEGs between PV+ interneurons in control and conditional homozygous *Ppargc1a* mice at P21. A threshold at $log_2FC > \pm 0.25$ was set. Genes enriched in SST+ interneurons are highlighted in blue.

(B) GFP, *Sst*, and *Calb1* expression in coronal sections through S1 of control and conditional homozygous *Ppargc1a* mice at P21.

(C) Density of GFP+ *Calb1*+ *Sst*− cells in L5 and L6 of S1 in control and conditional *Ppargc1a* mutant mice at P21 and P12.

(D) Expression changes (average log_2FC) in PV+ interneurons between control and conditional *Ppargc1a* mutant mice at P21 for genes enriched in PV+ interneurons or SST+ interneurons. A threshold at $log_2FC > \pm 0.25$ was set. Significant changes in expression are highlighted in magenta and blue for genes enriched in PV+ and SST+ interneurons, respectively.

(E) Expression changes (average log_2FC) in PV+ interneurons between control and conditional *Ppargc1a* mutant mice at P21 for genes enriched in the five main subclasses of cortical interneurons. A threshold at $log_2FC > \pm 0.25$ was set. Significant changes in expression are highlighted in magenta, dark blue, dark green, green, and orange for genes enriched in *Pvalb*, *Sst*, *Sncg*, *Vip*, and *Lamp5* subclasses of interneurons, respectively.

(F) PV and WFA expression in coronal sections through S1 of control, conditional heterozygous, and conditional homozygous *Ppargc1a* mice at P60.

(G) Density of GFP+ PV+ cells and GFP+ WFA+ cells across all layers of S1 in control, conditional heterozygous, and conditional homozygous *Ppargc1a* mice at P60.

Data are presented as mean ± SEM. Scale bar, 100 μm (B and F). *$p < 0.05$, **$p < 0.01$, ***$p < 0.001$. See Table S5 for details on statistics.

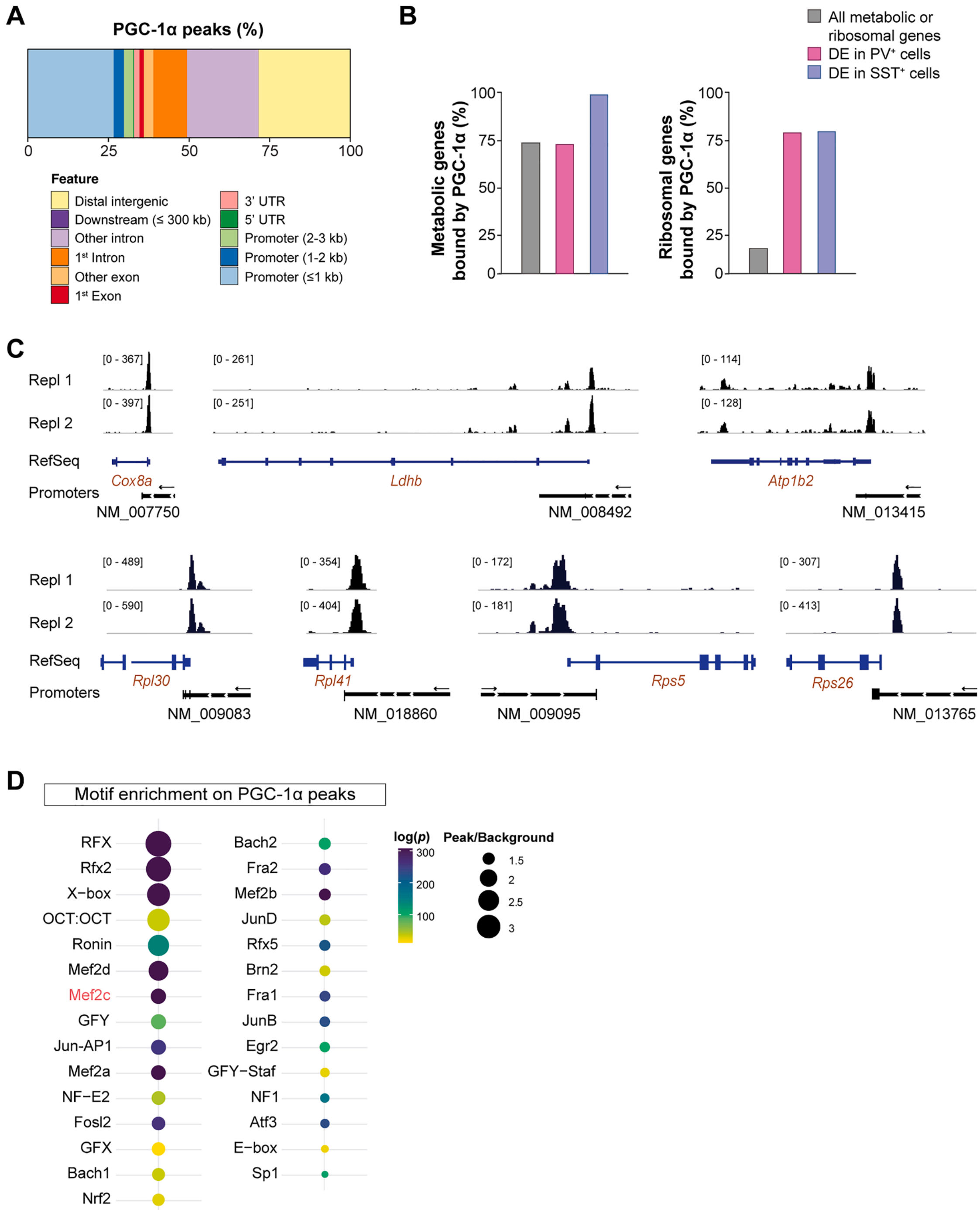

Figure S7. PGC-1α regulates gene expression through direct transcriptomic regulation, related to Figure 5
(A) Proportion of PGC-1α peaks observed on the different gene features (distal intergenic, introns, exons, UTR, and promoters).
(B) Plots depicting the proportion of all metabolic or all ribosomal genes directly bound by PGC-1α (gray) and the proportion of metabolic or ribosomal DEGs between control and conditional homozygous *Ppargc1a* PV+ cells (magenta) and SST+ cells (blue) directly bound by PGC-1α.
(C) Genome browser view of the genomic locus of metabolic (*Cox8a, Ldhb*, and *Atp1b2*) and ribosomal (*Rpl30, Rpl41, Rps5*, and *Rps26*) genes. The black peaks represent the read coverage following PGC-1α CUT&RUN in two replicates at P12. Gene promoters and the size scale of the peaks are annotated. The arrows above the promoters indicate the direction of transcription.
(D) Motif enrichment analysis at the site of PGC-1α peaks, revealing potential binding partners of PGC-1α to regulate gene expression. Peak/background represents the incidence of the motif among target peaks over the incidence among background sequences.

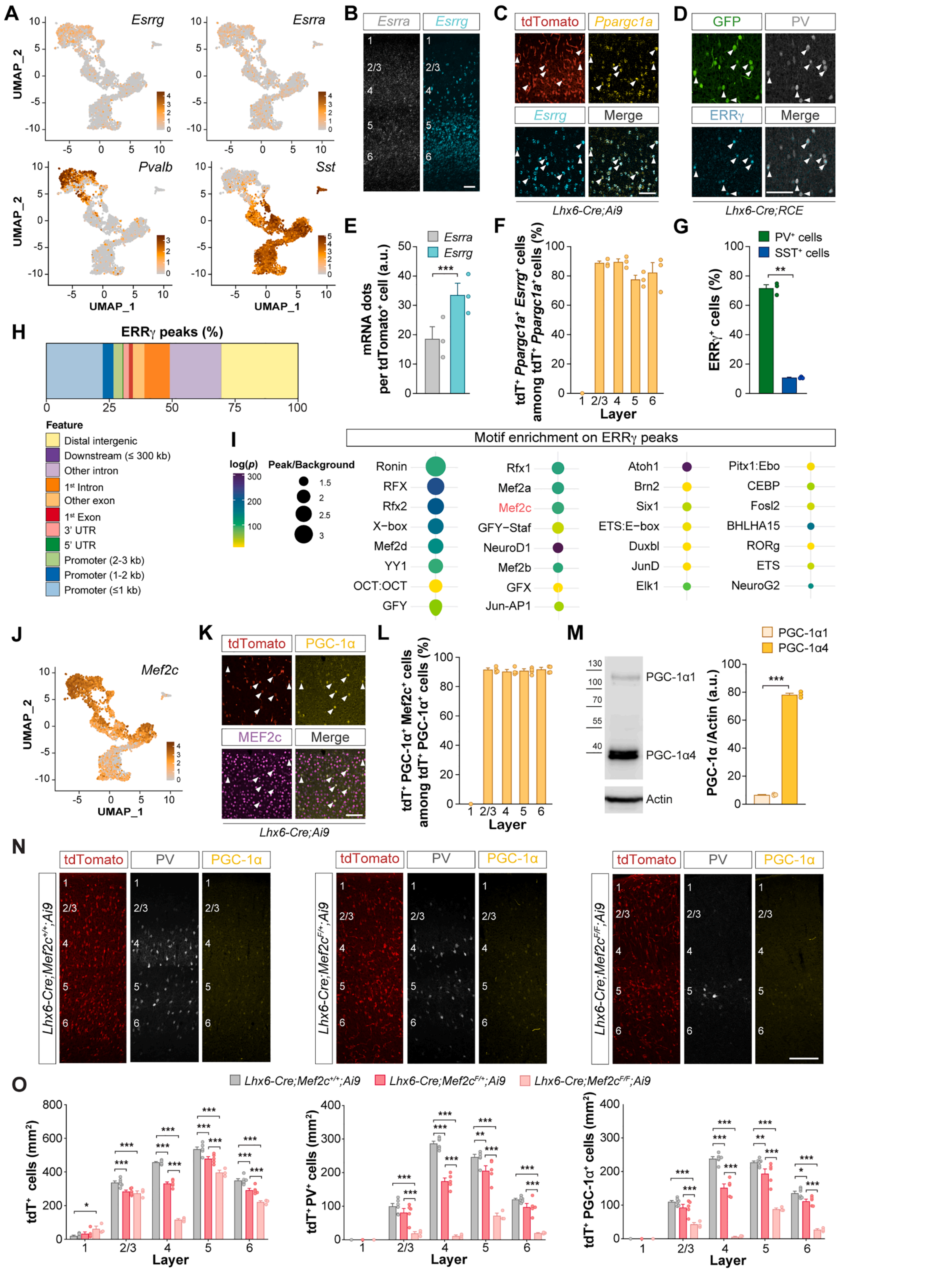

(legend on next page)

CellPress
OPEN ACCESS

Figure S8. Expression of *Esrrg*, ERRγ, and Mef2c in MGE-derived interneurons as partners and regulators of PGC-1α, related to Figure 6

(A) UMAP plots illustrating the expression of *Esrrg*, *Esrra*, *Pvalb*, and *Sst* in MGE-derived cells at P21.

(B) *Esrra* and *Esrrg* expression in coronal sections through S1 of *Lhx6-Cre;Ai9* mice at P12.

(C) *Esrrg* and *Ppargc1a* expression in tdTomato$^+$ cells in S1 of *Lhx6-Cre;Ai9* mice at P12.

(D) ERRγ expression in L2-4 GFP$^+$ PV$^+$ interneurons and prospective SST$^+$ (GFP$^+$ PV$^-$) interneurons in S1 of *Lhx6-Cre;RCE* mice at P21.

(E) Number of *Esrra* and *Esrrg* RNAscope dots present in tdTomato$^+$ cells in S1 L2-4 of *Lhx6-Cre;Ai9* mice at P12.

(F) Proportion of tdTomato$^+$ *Ppargc1a*$^+$ cells expressing *Esrrg* at P12 through cortical layers of S1 of *Lhx6-Cre;Ai9* mice.

(G) Proportion of PV$^+$ and prospective SST$^+$ cells expressing ERRγ across all layers of S1 in *Lhx6-Cre;RCE* animals at P21.

(H) Proportion of ERRγ peaks observed on the different gene features (distal intergenic, introns, exons, UTR, and promoters).

(I) Motif enrichment analysis at the site of ERRγ peaks, revealing potential binding partners of ERRγ to regulate gene expression. Peak/background represents the incidence of the motif among target peaks over the incidence among background sequences.

(J) UMAP plot illustrating the expression of *Mef2c* in MGE-derived cells at P21.

(K) MEF2c and PGC-1α expression in tdTomato$^+$ cells in S1 of *Lhx6-Cre;Ai9* animals at P12.

(L) Proportion of tdTomato$^+$ PGC-1α$^+$ cells expressing MEF2c at P12 through cortical layers of S1 in *Lhx6-Cre;Ai9* animals.

(M) Western blot image and quantification of the levels of expression of the short (PGC-1α4) and long (PGC-1α1) PGC-1α isoforms in S1 at P12.

(N) PV, PGC-1α, and tdTomato expression in coronal sections through S1 of control, conditional heterozygous, and conditional homozygous *Mef2c* mutant mice at P12.

(O) Density of tdTomato$^+$ cells, tdTomato$^+$ PV$^+$ cells, and tdTomato$^+$ PGC-1α$^+$ cells in S1 of control, conditional heterozygous, and conditional homozygous *Mef2c* mice at P12 across all cortical layers.

Data are presented as mean ± SEM. Scale bars, 10 μm (C, D, and K) and 100 μm (B and N). *$p < 0.05$, **$p < 0.01$, ***$p < 0.001$. See Table S5 for details on statistics.
