# Supplementary material for: A postnatal molecular switch drives activity-dependent maturation of parvalbumin interneurons
Source: Cell. Author manuscript; Available in PMC 2026 Oct 9. (PMC7619535; doi:10.1016/j.cell.2025.06.029)
Supplement: Supplementary file 2 [file EMS219678-supplement-Supplemental_information.pdf]

Cell, Volume *188*

Supplemental information

A postnatal molecular switch drives activity-dependent maturation of parvalbumin interneurons

Monika Moissidis, Leyla Abbasova, Martijn Selten, Rafael Alis, Clémence Bernard, Yaiza Domínguez-Canterla, Fazal Oozeer, Shenyue Qin, Audrey Kelly, Laura Mòdol, Navneet A. Vasistha, Benjamin Jones, Pawan Dhami, Konstantin Khodosevich, Fursham Hamid, Paul Lavender, Nuria Flames, and Oscar Marín

Table S1. Intrinsic electrophysiological properties of PV^+ interneurons in different experimental conditions, related to Figures 3 and S1.

**L5 PV+ interneurons in S1 of G42 mice** (Fig S1E)	**P12**	**P13**	
	[average ± S.E.M.]	[average ± S.E.M.]	p-value
**RMP (mV)**	-61.87 ± 1.23	-67.70 ± 1.06	0.002
**IR (MΩ)**	143.73 ± 13.53	125.70 ± 13.37	0.36
**Rheobase (pA)**	140.88 ± 18.65	200.11 ± 15.98	0.03
**Capacitance (pC)**	78.29 ± 10.49	73.64 ± 6.75	0.71
**Ih sag (mV)**	4.61 ± 0.59	4.87 ± 0.83	0.80
**Threshold (mV)**	-36.31 ± 0.91	-34.19 ± 1.25	0.20
**Half-width (ms)**	0.670 ± 0.033	0.598 ± 0.026	0.10
**Rise time (20-80, ms)**	0.196 ± 0.012	0.177 ± 0.007	0.19
**Decay time (80-20, ms)**	0.359 ± 0.016	0.322 ± 0.017	0.13
**AP Max (mV)**	27.74 ± 1.25	25.08 ± 1.05	0.12
**fAHP (min value, mV)**	-56.95 ± 0.79	-55.01 ± 0.93	0.13
**MFF (Hz)**	116.44 ± 12.77	141.50 ± 14.18	0.21
**N cells**	9	10	

**L2/3 PV+ interneurons in S1 at P21** (Fig 3G)	***Lhx6-Cre;Ppargc1a***$^{+/+}$	***Lhx6-Cre;Ppargc1a***$^{F/F}$	
	[average ± S.E.M.]	[average ± S.E.M.]	p-value
**RMP (mV)**	-65.46 ± 1.10	-66.74 ± 1.72	0.553
**IR (MΩ)**	78.07 ± 6.19	117.16 ± 7.14	0.0004
**Rheobase (pA)**	242.61 ± 21.59	192.05 ± 14.56	0.058
**Capacitance (pC)**	118.01 ± 17.75	88.00 ± 3.25	0.086
**Ih sag (mV)**	3.19 ± 0.30	2.20 ± 0.19	0.009
**Threshold (mV)**	-33.77 ± 0.74	-30.38 ± 1.177	0.028
**Half-width (ms)**	0.401 ± 0.014	0.520 ± 0.028	0.0013
**Rise time (20-80, ms)**	0.138 ± 0.004	0.189 ± 0.011	0.0005
**Decay time (80-20, ms)**	0.200 ± 0.007	0.248 ± 0.013	0.004
**AP Max (mV)**	26.34 ± 1.16	25.78 ± 1.11	0.729
**fAHP (min value, mV)**	-57.67 ± 0.54	-56.59 ± 1.00	0.375
**MFF (Hz)**	242.25 ± 11.43	165.57 ± 13.66	0.0003
**N cells**	12	14	

**L2/3 PV+ interneurons in S1 at P21** (Fig 3H)	***Gfp***	***Gfp + Ppargc1a* overexpression**	
	[average ± S.E.M.]	[average ± S.E.M.]	p-value
**RMP (mV)**	-66.67 ± 2.66	-63.97 ± 1.96	0.42
**R input (MΩ)**	99.88 ± 12.30	96.01 ± 11.00	0.82
**Rheobase (pA)**	226.61 ± 23.93	211.57 ± 27.00	0.70
**Capacitance (pC)**	71.68 ± 8.24	70.93 ± 6.11	0.94
**Ih sag (mV)**	3.43 ± 0.85	4.32 ± 0.60	0.39
**Threshold (mV)**	-32.48 ± 1.03	-33.45 ± 1.31	0.60
**Half-width (ms)**	0.511 ± 0.029	0.439 ± 0.014	0.028
**Rise time (20-80, ms)**	0.166 ± 0.009	0.145 ± 0.004	0.033
**Decay time (80-20, ms)**	0.248 ± 0.019	0.210 ± 0.008	0.058
**AP Max (mV)**	23.57 ± 1.93	25.76 ± 1.06	0.30
**fAHP (min value, mV)**	-57.70 ± 1.41	-59.36 ± 1.38	0.43
**MFF (Hz)**	181.43 ± 13.34	225.40 ± 13.65	0.042
**N cells**	7	10	

RMP, Resting Membrane Potential; IR, Input Resistance; Ih, h-current; AP, Action Potential; fAHP, fast Afterhyperpolarization; MFF, Maximum Firing Frequency.
